# The Effects of Sex-Type, the Sex of the Avatar, and Salience of the Sex of the Avatar on Emotional Valence and Arousal

**DOI:** 10.3389/fpsyg.2021.659547

**Published:** 2021-05-10

**Authors:** Duncan V. Prettyman, Paul D. Bolls

**Affiliations:** ^1^College of General Education and Psychology, Colorado Technical University, Colorado Springs, CO, United States; ^2^The Edward R. Murrow College of Communication, Washington State University, Pullman, WA, United States

**Keywords:** avatars, arousal, valence, psychophyisology, gender, sex, salience

## Abstract

The objective of this study was to investigate the effects of avatar sex, salience of avatar sex, and player sex-type on less conscious embodied emotional arousal and valence vs. consciously perceived emotional arousal and valence elicited by a gaming experience. The experiment conducted a 2 avatar sex (female × male) × 2 salience of avatar sex (high × low) × 2 player sex-type (sex-typed × non-sex-typed) mixed model factorial design. Participants were randomly assigned to one of two gameplay conditions (high-salience male and female avatar or low-salience male and female avatar) and then played two 15-min sessions of a video game—one session playing the game as a male avatar and one session playing the game as a female avatar. The order in which participants played as either a male or female avatar was randomized. Psychophysiological indicators of arousal (skin conductance) and valence (facial electromyography) were recorded during gameplay. Self-report measures of arousal and valence were obtained immediately after each 15-min session of gameplay. Data analysis tested hypotheses concerning the effects of avatar sex, salience of avatar sex, and player sex-type separately on real-time embodied variation in arousal and valence as revealed through physiological indicators and conscious perception of arousal and valence obtained through self-report measures.

## Introduction

According to the Entertainment Software Association (2019) 65% of American adults play video games and 75% of American households have at least one member who plays video games. The number of men and women playing video games is roughly equivalent and the average gamer is 33 years old (Entertainment Software Association, 2019). Video games have become a prevalent and popular form of entertainment over the last three decades, enjoyed by adults and children of all sexes and genders. As the number of video games fans continues to grow, research on how specific game features, like avatars, affect players' gaming experiences becomes increasingly important.

The entertainment experience of playing video games, like most forms of entertainment media, is rooted in emotional processes, like emotional arousal (the strength of the emotion) and emotional valence (whether the emotion is positive or negative; Bradley and Lang, 2007; Potter and Bolls, 2012), and responses (Lang, 2009; Fisher et al., 2018; Raney and Bryant, 2019). Previous research suggests that emotional processes and responses elicited by features of video games (e.g., point of view) can vary by gender as well as the nature of the observed emotional process (Lim and Reeves, 2009). The nature of the observed emotional process can vary according to the level of consciousness at which the response occurred (i.e., conscious or subconscious) and whether the process is observed in real time (as is the case with physiological indicators of emotion) or after playing a game (Lim and Reeves, 2009). The widespread appeal of video games along with potential variation in the nature of emotional processes and responses to video games makes it particularly important to understand how users of different gender types emotionally process games and what factors within games impact that processing (Lonergan et al., 2018).

The objective of this study was to investigate how the sex of the avatar (SOTA) (i.e., the digital bodies that represent users in virtual environments), the salience of the SOTA (i.e., the degree to which a player is consciously aware of the sex of their avatar), and player sex-type affect the embodied emotional processing of video games and conscious emotional responses to the gaming experience. Specifically, we investigated how less conscious these factors affected the embodied processes of emotional arousal and emotional valence (i.e., emotional arousal and valence) and how they affect conscious perceptions of emotional arousal and emotional valence (i.e., conscious emotional arousal and valence).

This project approached the question of how the SOTA, salience of the SOTA, and participant sex-type impact players' conscious emotional arousal and valence and less conscious emotional arousal and valence from several theoretical perspectives. Specifically, it draws on insights from gender schema theory (GST) and the limited capacity model of motivated mediated message processing (LC4MP).

The LC4MP is a model of information processing (Lang, 2009). According to the LC4MP, humans are limited-capacity processors, which means that they only have a limited number of cognitive resources to dedicate to the processing of information at any given time. Information processing is made up of three “simultaneous and continuous” subprocesses: (1) encoding (taking in information), (2) storage (storing encoded information in long-term memory), and (3) retrieval (pulling information out of memory; Lang, 2009, p. 194). The allocation of cognitive resources to these subprocesses is both controlled and automatic. Controlled allocation of cognitive resources refers to when a person purposefully allocates their resources to a specific subprocess or subprocesses, such as when he or she is trying to pay attention or remember something. The automatic allocation of cognitive resources occurs when cognitive resources are allocated in response to the activation of a person's motivational systems by “stimulus properties or in unconscious support of the conscious goals of the user” (Lang, 2009, p. 195). The LC4MP maintains that people have two motivational systems called the aversive system (i.e., the avoid system), which is associated with negative stimuli, and the appetitive system (i.e., the approach system), which is associated with positive stimuli. The motivational systems are independent and can be active at the same time. Which system is active, along with the strength of that activation, impacts how cognitive resources are allocated. According to the LC4MP, activation of the appetitive system causes more resources to be allocated to encoding and storage, while activation of the aversive system leads to an increase in resources to encoding followed by a slight decrease in resources dedicated to encoding. The stronger the activation of the system, the greater its effects on resource allocation. According to the LC4MP, people's emotions are directly tied to the activation of the motivational system; emotional valence represents which motivational system is active (i.e., appetitive vs. aversive), and emotional arousal represents the strength of the activation of the system(s). For example, a picture of a mutilated corpse would cause a strong activation of the aversive system because it is a very negative stimulus. In the context of the present study, LC4MP acts as a framework for understanding and explaining how the sex of an avatar can affect players' conscious perceptions and less conscious embodied processes of emotional arousal and valence. The idea is that the SOTA is a stimulus feature that is motivationally relevant and thus can activate a person's motivational systems. To understand why the SOTA may be a motivationally relevant stimulus feature we must turn to the second major theory used in this study, GST.

A schema is a mental network of information related to a particular topic/object/issue/etc. that guides a person's perceptions (Bem, 1981b; Martin and Halverson, 1981). Gender schema theory maintains that people form schemas about “themselves and the sexes,” called gender schemas, that guide their perceptions and that influence how they process information and behave (Martin et al., 2002, p. 911). When a person is presented with new information, they will process that information based on what is contained in their schema related to that information; this is known as “schematic processing” (Bem, 1981b). For example, if your gender schema included the idea that dresses are only for women, then when you see a person in a dress you would likely assume that the person is a woman. Information that is schematic (i.e., fits with a person's schema) is processed more easily because a mental structure already exists to process it, whereas information that is a-schematic (i.e., does not fit with a person's schema) is not processed as easily and requires more effort. According to GST, for certain people the gender identity associated with their biological sex is so central to their self-concept that they process all, or nearly all, information through the lens of their gender schema; these people are called “sex-typed” (Bem, 1981b). Individuals for whom the gender identity associated with their biological sex is not as central to their self-concept are called “non-sex-typed” and are less likely to process information in terms of their gender schema (Bem, 1981b). With regards to the present study, the ideas of gender schemas, schematic processing, and sex-typing can be used to explain how the SOTA can be a motivationally relevant cue that can affect individuals' emotional arousal and valence.

The basic idea is that people's schemas influence what is motivationally relevant to them or not. Information that fits within our pre-existing schemas (i.e., schematic information) should be more motivationally relevant than information that does not fit into our pre-existing schemas (i.e., a-schematic information). Previous research has established that schematic information leads to increased conscious perceptions of emotional arousal and positive emotional valence (Shapiro et al., 2002; Fujioka, 2005). Because conscious perceptions of emotional arousal and valence tend to mirror less conscious embodied processes of emotional arousal and valence (Greenwald et al., 1989), it is reasonable to suggest that schematic information has similar effects on embodied processes of emotional arousal and valence. Thus, schematic information should lead to increased emotional arousal and increased positive valence.

With regards to the present study, this means that (1) when sex-typed players use an avatar with a sex that matches their gender identity (e.g., male avatar with masculine gender) it should lead to higher emotional arousal and positive emotional valence compared with when they use an avatar that does not match their gender identity (e.g., male avatar with feminine gender) and (2) that non-sex-typed players' conscious perceptions and embodied processes of emotional arousal and valence should not differ, regardless of the sex of their avatar. Because sex-typed players are constantly processing information in terms of their gender schemas, when the SOTA matches their gender identities (i.e., is schematic) it is motivationally relevant and thus causes increased emotional arousal and valence; however, when the SOTA does not match with their gender identities (i.e., is a-schematic), it is not motivationally relevant and thus does not affect emotional arousal and valence. Additionally, because non-sex-typed players do not use their gender schemas as the basis for processing information, the SOTA should not be motivationally relevant to them regardless of whether or not it matches their gender identity, which means it should have no effect on their emotional arousal and valence.

However, there is reason to believe that same-sex avatars and opposite-sex avatars may affect sex-typed individuals' conscious perceptions and embodied processes of emotional arousal and valence differently. According to GST, in addition to possessing a readiness to process information in terms of their gender schemas, sex-typed individuals have a “generalized readiness to encode all cross-sex interactions in sexual terms” (Bem, 1981b, p. 361). This would suggest that for sex-typed people avatars of the opposite sex should be more motivationally relevant than avatars of the same sex. In other words, for sex-typed players an avatar of the opposite sex should be more motivationally relevant, and thus lead to higher emotional arousal and valence, than one of the same sex because the opposite-sex avatar is more relevant to basic survival needs (i.e., the need for procreation/mates Lang, 2009; Fisher et al., 2018).

These two differing sets of predictions can be reconciled when they are considered in relation to people's conscious and unconscious motivations/goals as described in the LC4MP. The predictions based on sex-typed individuals' tendency to process cross-sex interactions in sexual terms may reflect their subconscious goals, whereas those made based on their readiness to process all information in terms of their gender schema may be more reflective of sex-typed individuals' conscious goals. In the context of the present study, this means that it is likely that sex-typed participants' self-reported arousal and valence and their arousal and valence as indicated by psychophysiological measures will not match. Specifically, that when sex-typed participants' gender identities and the sex of their avatar match, they will report greater arousal and emotional valence then when using an avatar whose sex does not match their gender identity, while sex-typed participants' arousal and valence as indicated by physiological measures should do the opposite (i.e., when gender identity and avatar sex mismatch, arousal and valence would be higher than when using an avatar with a matching sex).

Unlike self-report measures, which are by their nature reliant on an individual's conscious perceptions, psychophysiological measures provide a way to see inside the “black box” of the human mind and capture some evidence of psychological phenomena, such as embodied processes of emotional arousal and valence, that participants may not be consciously aware of (Lang et al., 2009; Potter and Bolls, 2012). Because the predictions made based on sex-typed individuals' tendency to process cross-sex interactions in sexual terms are more concerned with “subconscious” motivations, we believe that the psychophysiological measures of emotional arousal and valence will better capture those predictions and that the predictions made based on sex-typed individuals' readiness to process all information in terms of their gender schema, which are more concerned with conscious motivations, will be best captured in the self-report measures of emotional arousal and valence.

So far we have discussed the effect that the sex of a player's avatar will have on his or her emotional arousal and emotional valence; however, we have not discussed the impact that the salience of the SOTA will have on either of these variables. The salience of the SOTA is particularly important as there is reason to believe that none of the above effects will be seen unless the sex of a player's avatar is made salient to them.

Video games are complex stimuli containing a plethora of characteristics that can all affect an individual's emotional arousal and valence (audio, video, haptic feedback, etc.). According to the LC4MP, individuals are most affected by message features and content that are motivationally relevant to them (Lang, 2009; Fisher et al., 2018). There are a number of message features and content types that are universally motivationally relevant because they are tied to humans' evolutionary past (e.g., graphic depictions of violence, opposite-sex nudes, smiling babies, etc.). Much of the content in video games falls into this category of “survival needs” (e.g., explosions, enemies, weapons, hazardous environments, etc.) and because of this is likely prioritized over other content/features, such as the SOTA^1^. Due to the prioritization of survival-need-related content, the influence of other factors in a video game that could affect an individual likely go unnoticed by the brain because they are not as motivationally relevant. Put another way, when you are attempting to defeat a horde of hostile robots while simultaneously escaping an exploding space station, the sex of your avatar is likely the furthest thing from your mind.

Previous work by Coyle and Liben (2016) suggests that when the salience of the SOTA is low (i.e., players are not consciously aware of it) it has no effects on people, but that when the salience of the SOTA is high (i.e., players are consciously aware of it) it does have effects. Specifically, they found that avatars with low sex salience had no effects on either sex-typed or non-sex-typed individuals, and that avatars with high sex salience influenced sex-typed people, but had no effects on non-sex-typed people (Coyle and Liben, 2016).

In the context of the present study this means that although the SOTA could have an influence on sex-typed individuals' emotional arousal and valence, because of everything else going on in the game any effect that the SOTA has on their emotional arousal and valence is likely “washed out.” However, if the sex of sex-typed players' avatars were made more salient to them (i.e., brought to the fore of their thoughts), then it is possible that it would affect their emotional arousal and valence in the manner predicted above. The idea is that when the salience of the SOTA is low, sex-typed players are not consciously aware of the sex of their avatars. If sex-typed players are not consciously aware of the sex of their avatars, then the SOTA is less likely to trigger gender schematic processing, and thus it is less likely to be motivationally relevant. However, when the salience of the SOTA is high, sex-typed players are consciously aware of the sex of their avatars. If sex-typed players are consciously aware of the sex of their avatars, it is likely to trigger gender schematic processing, and thus have motivational relevance. In other words, how salient the sex of sex-typed players' avatars is to them may determine whether or not the effects of the sex of their avatars on their conscious perceptions and embodied motivational processes of emotional valence and arousal are masked by the effects of the rest of a game's content. In the context of the present project this means that in addition to testing the effects of the SOTA on sex-typed and non-sex-typed individuals' conscious perceptions and embodied processes of emotional arousal and valence, the effects of the salience of the avatar's sex will also be tested.

Prior to formally laying out our research questions and hypotheses, we will briefly review the argument being made. Gender schema theory predicts that people will process information differently based on their sex-type. Specifically, that when the SOTA matches their gender identity for sex-typed people it will be schematic, and thus motivationally relevant; and, that when the SOTA does not match their gender identity it will be a-schematic, and thus not motivationally relevant. Additionally, the SOTA is neither schematic nor a-schematic for non-sex-typed people and thus has no motivational relevance to them. According to the LC4MP, motivationally relevant content features can affect individuals' conscious perceptions and embodied processes of emotional arousal and valence. Thus, the SOTA will differentially affect individuals' conscious perceptions and embodied processes of emotional arousal and valence based on their sex-type. Additionally, it is argued that in order for the SOTA to have any effects on individuals, regardless of their sex-type, it must be salient to them. If the sex of players' avatars is not salient to them, then the SOTA will have no effect on conscious perceptions and embodied processes of emotional arousal and valence. However, when the salience of the sex of players' avatars' is high, it will effect conscious perceptions and embodied processes of emotional arousal and valence based on the sex-type of the player.

The specific predicted effects for conscious emotional responses and less conscious embodied emotional processing are presented in the following hypotheses:^2^

H1: Participants' conscious (a) arousal and (b) valence will not differ significantly between low-salience male and low-salience female avatar conditions, regardless of sex-typing.

H2: Sex-typed participants' conscious (a) arousal and (b) valence will be higher for high-salience avatars of the same-sex vs. the opposite-sex.

H3: Non-sex-typed participants' conscious (a) arousal and (b) valence will not differ significantly between the high-salience avatars of both sexes.

H4: Participants' subconscious (a) arousal and (b) valence will not differ significantly between low-salience male and low-salience female avatar conditions, regardless of sex-typing.

H5: Sex-typed participants' subconscious a) arousal and b) valence will be higher for opposite-sex avatars than for same-sex avatars.

For non-sex-typed people it is unclear how the SOTA will affect their embodied motivational processes of emotional arousal and valence when the salience of the SOTA is high. On the one hand, previous research has suggested that player-avatar similarity can impact players' identification with their avatars (Trepte and Reinecke, 2010). When the SOTA is highly salient to non-sex-typed people, if its sex matches their biological sex, it may contribute to their feelings of player-avatar similarity and thus be motivationally relevant. If this was the case, then non-sex-typed people using same-sex avatars would lead to increased embodied processes of emotional arousal and valence. However, because non-sex-typed people do not generally process information in terms of their gender schema, it is unlikely that the sex of their avatar would have any impact on their conscious perceptions of emotional arousal and valence. But, it is also possible that for non-sex-typed individuals there will be no significant differences between embodied motivational processes of emotional arousal and valence. The reasoning is that either both the male and female avatars will be equally motivationally relevant, thus leading to no significant differences between either condition; or neither of the avatars (male or female) is motivationally relevant, and thus leads to no significant differences between the conditions. Therefore, we ask:

RQ1: How are non-sex-typed participants' embodied motivational processes of emotional arousal and emotional valence affected by an avatar with a highly salient sex?

## Materials and Methods

In order to test the above hypotheses and answer the research questions, a mixed-design experiment was performed.

### Design

The experiment conducted utilized a 2 avatar sex (female × male) × 2 salience of avatar sex (high × low) × 2 sex-type (sex-typed × non-sex-typed) mixed model factorial design. Avatar sex was a within-subject factor, and salience of avatar sex, sex-type, and order served as between-subject factors. Avatar sex consisted of two levels, male and female. Salience of avatar sex consisted of two levels, high and low. Sex-type consisted of two levels, sex-typed and non-sex-typed. The non-sex-typed level consisted of both androgynous and undifferentiated individuals. Previous research has shown that androgynous and undifferentiated individuals' responses to and processing of gender information is similar with few exceptions (Bem, 1981b; Markus et al., 1982). The order factor represents the order in which subjects experienced the stimuli (i.e., male avatar then female avatar or female avatar then male avatar). Subjects were randomly assigned to one of the two order conditions to control for any order effects, participant fatigue, and desensitization.

### Power Analysis

To determine the necessary sample size, a power analysis for a repeated measures ANOVA with a within-between interaction, a Cohen's *f* of 0.18, an α of 0.05, an expected power of 0.80, three groups, two measurements, a correlation among repeated measures of 0.5, and a non-spherecity correction of 1 was conducted using GPower 3.1, which indicated a minimum sample size of 78 participants. A small effect size was chosen as previous research using psychophysiological measures has indicated that the effect size for content features on embodied processes of emotional arousal and valence tend to be small (Potter and Bolls, 2012).

### Sample

A sample of 106 male and female students from a large southern university in the USA was used for the study. Participants were recruited using a research participant pool and received extra credit for their participation. Of the 106 participants, 13 were removed from the sample due to procedural issues during data collection, and another 12 were removed due to excessive artifacts in their skin conductance data, leaving a final sample size of 81 participants. The final sample used for analysis was predominately female (55.6%, *n* = 45; male 44.4%, *n* = 36) and white (66.7%, *n* = 54; African American 12.3%, *n* = 10; American Indian or Alaska Native 1.2%, *n* = 1; Asian 4.9%, *n* = 4; Other 14.8%, *n* = 12).

### Stimulus

The stimulus used for the study was the roleplaying game (RPG) *The Elder Scrolls V: Skyrim* by Bethesda Game Studios (2011). *The Elder Scrolls V: Skyrim* is an action-RPG in which players explore a fantasy world defeating monsters, collecting magic items, and completing quests for experience points. In this study, participants played through the “Shimmermist Cave” dungeon. The Shimmermist Cave dungeon is a circular cave system that contains several non-human enemies, a few traps, and a few treasure chests; it is recommended for characters of level 18 or higher (Skyrim: Shimmermist Cave, 2018). Shimmermist Cave was chosen for this study for several reasons. First, it is circular and entirely self-contained; the last room of the dungeon connects back to the entrance area of the dungeon to create a loop. By being self-contained Shimmermist Cave allows participants to have relatively similar gameplay experiences. Second, Shimmermist Cave contains a number of treasure chests, which are important for the task participants were asked to complete in the gameplay portion of the experiment. For the gameplay portion, participants were asked to locate six treasure chests in Shimmermist Cave, one more than actually exists in the cave. This task was intended as a way to occupy participants for the full 15 min of the gameplay section; because the dungeon is circular, even if participants completed it in under 15 min, they ended up back at the beginning of the dungeon and could continue exploring the dungeon as they looked for the “sixth” treasure chest. Third, Shimmermist Cave contains only monster enemies. Using a dungeon that only contains monster enemies avoided introducing any potential confounds from encountering other humanoid avatars during gameplay. Additionally, the enemies in Shimmermist Cave scale to some degree to match the level of the player (Skyrim: Shimmermist Cave, 2018). In the current study players used a level 81 character and were made invincible (i.e., they could not be killed by enemies) in order to control for varying levels of gaming skill. Participants were not informed of their invincibility in order to maintain natural reactions to in-game events.

### Procedure

Upon arriving at the laboratory, participants were greeted and given an informed consent form to complete. Participants were told that they were participating in preliminary research for a game developer that the university had partnered with and that the developer was interested in getting feedback on character models used in their games. They were also told that because the research was preliminary what they tested may or may be part of an existing game. This information was described on the informed consent form and given verbally to the participants. After completing the informed consent form, participants were taken to the data collection room. They were seated in front of a computer on which they completed a pre-test questionnaire that contained demographic questions, the Bem Sex Role Inventory (BSRI), and several other questions not analyzed as part of the present project. After participants had completed the pre-test questionnaire, the researcher prepped participants' skin for sensor placement and then placed sensors on participants for the collection of facial electromyography (fEMG) and skin conductance (SC) data. After all the sensors had been placed, the researcher gave participants a sheet listing the game's controls and provided them with instructions on how to play the game. Next, participants watched a nature video and a 2-min baseline was taken of their fEMG and SC. Following baseline collection, the researcher returned to the participants and gave them instructions for the gameplay portion of the experiment. After the instructions were given, participants began the gameplay portion. Participants' gameplay was recorded using a webcam and synced to their physiological data via the Acqknowledge software.

During the gameplay portion of the experiment participants played the action-RPG *The Elder Scrolls V: Skyrim* on a widescreen computer monitor with a mouse and keyboard and headphones. The gameplay portion of the experiment consisted of two gameplay sessions; one using a male avatar and one using a female avatar. The order of the sessions (i.e., male then female, or female then male) was determined by participants' randomly assigned condition. The procedure for both sessions was identical save for the SOTA the player used. In each session participants played *The Elder Scrolls V: Skyrim* for 15 min, during which they had to explore a cave that contained various traps, enemies, and treasures. Participants were tasked with the goal of locating six different treasure chests in the dungeon and instructed to keep playing until they located all six chests or the 15 min expired. The path through the cave was relatively linear and made a large loop (i.e., it wrapped back to the starting area eventually). After 15 min, the researcher paused the game and participants completed an online questionnaire that contained self-report measures for arousal and valence and several distractor questions to help maintain the cover story.

Following administration of the questionnaire, participants completed a second gameplay session. The second gameplay session was identical to the first session except participants used a different avatar. Which avatar participants used during the second gameplay session was determined by their randomly assigned condition (e.g., if a participant played through the first session as the male avatar they would then complete the second session using the female avatar). Following the second session, participants completed another online questionnaire identical to the first one. Once participants had completed two gameplay sessions and the two online questionnaires, the researcher removed the sensors from them, thanked them, and dismissed them.

After all data collection was completed, an email was sent to all participants that debriefed them about the deception used in the study. Specifically, it informed participants that (1) the purpose of the study was to determine the effects of the sex of the player's avatar on their emotional arousal and emotional valence, (2) that the study was not part of a partnership between the university and an undisclosed game developer, (3) and that the number of chests in the level was actually five. The email also offered participants the opportunity to have their data withdrawn from use should they so desire now that the deception had been revealed. No participants requested to withdraw their data.

### Measures

#### Avatar Sex

The SOTA was conceptualized as whether an avatar is male or female. The SOTA was operationalized by using physical characteristics of the in-game models as well as data provided in the game about the avatars; specifically, whether the avatar was listed as male or female in the character creator. Avatar sex was manipulated by assigning participants to play *The Elder Scrolls V: Skyrim* using either a male or female avatar. In *The Elder Scrolls V: Skyrim* players can choose from a number of human races (e.g., Nord, Imperial, Redguard). For the present study the default male and female Imperial avatars were chosen. The Imperial race was chosen because it was the only race whose default male and female avatars did not feature face paint or other facial markings. The default male Imperial avatar is Caucasian with short black hair and a goatee (see Supplementary Figure 1). The default female Imperial avatar is Caucasian with shoulder length dirty-blonde hair and wears minimal makeup (see Supplementary Figure 1).

#### Salience of the Sex of the Avatar

The salience of the SOTA was conceptualized as the degree to which players are consciously aware of the sex of their avatars. The salience of the SOTA was operationalized as whether a player used a high-salience avatar or a low-salience avatar. The salience of the sex of a player's avatar was manipulated by equipping the avatar with different attire which obscured or emphasized the avatar's body shape. Evidence suggests that video game players determine whether an avatar is male or female based on a combination of social gender cues (e.g., hair length, gendered clothing) and how closely it conforms to the ideal male body shape or the ideal female body shape (Wade, 2012). In other words, one of the main ways that sex differences in video games are created is by emphasizing the differences that exist between male and female anatomy (e.g., breasts) and the presence of other gender cues (e.g., long hair).

In the high-salience condition the male and female avatars were equipped with the “ragged trousers” clothing item. The ragged trousers clothing item is a pair of well-worn, cloth, brown pants. When equipped to male avatars it leaves the wearer's upper torso exposed, and when equipped to female avatars a similarly styled shirt (i.e., ragged cloth) covers the upper torso. The game was modified so that the ragged trouser clothing item appeared identically on both the male and female avatars (i.e., it left the wearer's upper torso exposed). For male avatars this left them bare chested, and for female avatars it exposed a bikini-like bra (see Supplementary Figure 2). In the low-salience condition the male and female avatars were equipped with a full set of “ebony armor.” The ebony armor item consists of a bulky set of black plate armor that covers an avatar's body completely save for the head. The ebony armor looks nearly identical on both the male and female avatars (see Supplementary Figure 2).

#### Sex-Type

Participant sex-type was conceptually defined as how strongly individuals identify with the gender associated with their biological sex (Bem, 1981b). Participant sex-type was determined using participants' scores on the short form of the BSRI and their self-reported biological sex^3^. Following procedures outlined by Bem (1974, 1981a,b) participants were placed into one of three sex-type categories (i.e., sex-typed, non-sex-typed, or cross-sex-typed) based on their self-reported biological sex and their gender identity (i.e., masculine, feminine, androgynous, or undifferentiated) as determined by the BSRI^4^. Participants whose biological sex was reported as male or female and who were categorized as masculine or feminine by the BSRI, respectively, were categorized as sex-typed. Participants whose biological sex was reported as male or female and who were categorized as androgynous or undifferentiated by the BSRI were categorized as non-sex-typed. Participants whose biological sex was reported as male and categorized as feminine by the BSRI and participants whose biological sex was reported as female and categorized as masculine by the BSRI were both categorized as cross-sex-typed. Of the 81 participants 31 were sex-typed (38.3%), 30 were non-sex-typed (37%), and 20 were cross-sex-typed (24.7%).

Cross-sex-typed individuals were excluded from the analyses performed because none of the hypotheses proposed and tested included them. Research on the effect of cross-sex-typed people's gender schemas on their processing of information finds that it is inconsistent (Bem, 1981b). In some situations cross-sex-typed people process information in terms of their gender schemas and in other situations they do not (Bem, 1981b). Unlike sex-typed and non-sex-typed individuals who predictably process information in terms or not in terms of their gender schemas, cross-sex-typed individuals process information in terms of their gender inconsistently. Without being able to predictably determine when cross-sex-typed people would be processing information in terms of their gender schemas, and thus when the SOTA may or may not be motivationally relevant, no predictions could be made about how the SOTA or the salience of the SOTA would affect their conscious and less conscious emotional arousal and valence. Additionally, the distinct lack of consistency in the use of gender schematic processing among cross-sex-typed individuals prevents them from simply being grouped with sex-typed or non-sex-typed individuals who consistently use or do not use gender schematic processing.

#### Arousal

Arousal was conceptually defined as an indicator of the strength of activation in the motivational systems (Potter and Bolls, 2012). Conscious perceptions of arousal were operationalized as participants' self-reported scores on the arousal portion of the Self Assessment Manikin (SAM). The SAM is a validated 9-point pictorial scale used for assessing emotional arousal and valence (Lang, 1980; Bradley and Lang, 1994). For the arousal portion of the SAM, the scores range from 1 (low arousal) to 9 (high arousal). In addition to collecting participants' conscious perceptions of arousal, their subconscious processes of arousal as they unfolded across time were measured using physiological measures of arousal. Skin conductance has been found to be a good physiological indicator of arousal; thus, subconscious arousal was operationalized as participants' SC (Hopkins and Fletcher, 1994; Potter and Bolls, 2012). As SC increases arousal is said to increase, and as SC decreases arousal is said to decrease (Potter and Bolls, 2012). Skin conductance data were collected using a BioPac EDA 100C Electrodermal Activity Amplifier and disposable 8 mm Ag/AgCl electrodes. Participants' hands were cleaned with distilled water prior to electrode placement. Electrodes were placed on the palm of participants' left hand.

#### Valence

Valence was conceptualized as an indicator of which motivational system(s) are active (Potter and Bolls, 2012). Conscious perceptions of valence were operationalized as participants' scores on the valence portion of the SAM. The SAM is a validated 9-point pictorial scale for measuring emotional arousal and valence (Lang, 1980; Bradley and Lang, 1994). Valence scores on the SAM range from 1 (negative) to 9 (positive). In addition to collecting conscious perceptions of valence, participants' subconscious processes of valence as they unfolded across time were measured using physiological measures of valence. Subconscious valence was operationalized as facial muscle activity as indexed by fEMG. Increased muscle activity in the corrugator supercilii can indicate activation of the aversive system, and decreased muscle activity in the corrugator supercilii can indicate activation of the appetitive system (Cacioppo et al., 1986; Lang et al., 1993; Larsen et al., 2003). Facial electromyography data were collected using a BioPac EMG100C Electromyogram Amplifier and disposable 4 mm, Ag/AgCl floating electrodes. Prior to electrode placement, participants' skin was cleaned using a combination of makeup wipes and alcohol wipes and then conductive gel was applied. Electrode placement on the face followed the recommendations outlined in Potter and Bolls (2012) for fEMG data collection.

#### Time Spent Playing the Elder Scrolls V: Skyrim

In order to control for potential knowledge-based effects from previous experiences with *The Elder Scrolls V: Skyrim* participants were asked to indicate how many hours they had spent playing it in the last month on a 5-point Likert-type scale ranging from “0 to 10 h” to “41 or more hours.” Due the age (i.e., 8 years old at the time of writing) and popularity of *The Elder Scrolls V: Skyrim* there is a high chance that participants had played it previously (The Elder Scrolls V: Skyrim, 2019). Thus, in order to gain a clearer idea of participants' current knowledge of the game we measured how much time they spent playing the game recently. Of the 81 participants the vast majority had played 10 or less hours of *The Elder Scrolls V: Skyrim* in the past 30 days (80.2%, *n* = 65). The remaining participants had played the game anywhere from 11–20 h (11.1%, *n* = 9) to 21–30 h (1.2%, *n* = 1) to 31–40 h (1.2%, *n* = 1) to 41 or more hours (6.2%, *n* = 5) in the last 30 days.

#### Apparatus

Physiological measures were collected using a BioPac MP150 system with heart rate, skin conductance, and electromyography modules attached. BioPac's AcqKnowledge 5.0 software was used to control the physiological data collection. Participants' gameplay was recorded using a webcam and synced to their physiological data using AcqKnowledge 5.0.

## Results

### Physiological Data Editing and Reduction Procedures

Skin conductance data were collected at 20 Hz and then averaged over 1 s intervals across each 2-min baseline and each 15-min gameplay segment. Skin conductance data for the baselines and the gameplay segments were then down-sampled to 90 data points each, representing 10 s of gameplay. Following down-sampling, data were cleaned for artifacts. For SC, data were considered artifacts if a values were below 1. Artifacts were replaced with the next most likely neighbor. If 30% or more of a participant's data had to be replaced, their data were dropped from analysis (Potter and Bolls, 2012). The data from 12 participants were dropped from analysis due to excessive artifacts. Following data cleaning, change scores were calculated for each gameplay segment. Change scores (i.e., the change for a given score from the baseline) were calculated by first averaging each baseline from the 90 down-sampled data points from the 2 min of viewing the nature video, then subtracting that average from each of the 90 down-sampled gameplay segment data points. This process resulted in 90 SC change scores for each gameplay segment. All analyses of SC data were performed on the calculated change scores. Additionally, for all analysis of SC data, if Mauchly's Test of Sphericity was violated, significance results are reported as Greenhouse-Geisser tests.

Facial electromyography data (corrugator activity) was collected at 20 Hz. A Bandpass filter was applied to the data with a low frequency of 90 Hz and a high frequency of 500 Hz. Following application of the Bandpass filter, data were rectified. After the data were filtered and rectified, they were averaged over 1 s intervals across each 2 min baseline and each 15 min gameplay segment. Facial electromyography data for the baselines and the gameplay segments were then down-sampled down to 90 data points each, representing 10 s of gameplay. Following down-sampling, fEMG data were cleaned for artifacts. Artifacts were identified by looking at the range of responses within each individual for obvious outliers. Artifacts were replaced with the next most likely neighbor. If 30% or more of a participant's data had to be replaced, their data were dropped from analysis (Potter and Bolls, 2012). Following cleaning, change scores were calculated for each gameplay segment. Change scores were calculated by first averaging each baseline from the 90 down-sampled data points from the 2 min of viewing the nature video, then subtracting that average from each of the 90 down-sampled gameplay segment data points. This process resulted in 90 fEMG change scores for each gameplay segment. All analyses of fEMG data were performed on the calculated change scores. Additionally, for all analyses of fEMG data, if Mauchly's Test of Sphericity was violated, significance results are reported as Greenhouse-Geisser tests.

### Tests of Hypotheses

#### H1

Hypothesis 1 stated that participants' conscious (1) arousal and (2) valence would not differ significantly between the low-salience male and low-salience female avatar conditions, regardless of sex-type. To test both parts of this hypothesis, two separate two-way mixed design ANOVAs were calculated, one for arousal and one for valence.

A two-way mixed design ANOVA was calculated comparing participants' self-reported arousal scores for the low-salience male avatar and the low-salience female avatar. The SOTA (male × female) was entered as a within-subjects factor, and participant sex-type (sex-typed × non-sex-) was entered as a between-subjects factor. Missing values were excluded listwise. The main effect of the SOTA was not significant [*F*_(1, 24)_ = 0.000215, *p* = 0.988, η_*p*_^2^ = 0.000009]. The main effect of sex-type was also not significant [*F*_(1, 24)_ = 0.238, *p* = 0.630, η_*p*_^2^ = 0.010]. Lastly, the interaction of the SOTA and sex-type was not significant [*F*_(1, 24)_ = 0.515, *p* = 0.480, η_*p*_^2^ = 0.021]. Therefore, neither the SOTA nor a participants' sex-type had a significant effect on self-reported arousal in the low-salience condition (see Table 1).

**Table 1:** Means and standard deviations for self-reported arousal and valence for low-salience condition.

**Condition**	***M***	***SD***
**AROUSAL**		
**Male Avatar**		
Sex-typed (*n* = 16)	5.5625	2.22017
Non-sex-typed (*n* = 10)	4.9000	2.18327
**Female Avatar**		
Sex-typed (*n* = 16)	5.2500	1.98326
Non-sex-typed (*n* = 10)	5.2000	1.98886
**VALENCE**		
**Male Avatar**		
Sex-typed (*n* = 17)	5.1176	1.40900
Non-sex-typed (*n* = 9)	5.6667	2.29129
**Female Avatar**		
Sex-typed (*n* = 17)	5.0000	2.09165
Non-sex-typed (*n* = 9)	5.8889	2.08833

A two-way mixed design ANOVA was calculated comparing participants' self-reported valence scores for the low-salience male avatar and the low-salience female avatar. The SOTA (male × female) was entered as a within-subjects factor, and participant sex-type (sex-typed × non-sex-typed) was entered as a between-subjects factor. Missing values were excluded listwise. The main effect of the SOTA was not significant [*F*_(1, 24)_ = 0.031, *p* = 0.861, η_*p*_^2^ = 0.001]. The main effect of sex-type was also not significant [*F*_(1, 24)_ = 0.948, *p* = 0.340, η_*p*_^2^ = 0.038]. Lastly, the interaction of the SOTA and sex-type was not significant [*F*_(1, 24)_ = 0.331, *p* = 0.861, η_*p*_^2^ = 0.014]. Therefore, neither the SOTA nor a participants' sex-type had a significant effect on self-reported valence in the low-salience condition (see Table 1).

The results of both two-way mixed design ANOVAs suggest that participants' conscious (1) arousal and (2) valence did not significantly differ between the low-salience male avatar and low-salience female avatars regardless of sex-type. These findings are in-line with the predictions made by Hypothesis 1.

#### H2

Hypothesis 2 stated that sex-typed participants' conscious (1) arousal and (2) valence would be higher for high-salience avatars of the same-sex vs. high-salience avatars of the opposite sex. To test both parts of this hypothesis, two separate one-way ANOVAs were calculated, one for arousal and one for valence.

A one-way ANOVA was conducted comparing sex-typed participants' self-reported arousal for high-salience same-sex avatars to their self-reported arousal for high-salience opposite-sex avatars. Player-avatar sex match (same-sex × opposite-sex) was entered as a within-subject factor. Missing values were excluded listwise. There was no significant effect of player-avatar sex match on self-reported arousal [*F*_(1, 13)_ = 2.019, *p* = 0.179, η^2^ = 0.134]. This suggests that SOTA had no significant impact on self-reported arousal for sex-typed participants in the high-salience condition (see Table 2).

**Table 2:** Means and standard deviations for self-reported arousal and valence for sex-typed participants in the high-salience condition.

**Condition**	***M***	***SD***
**AROUSAL**		
Same-sex (*n* = 14)	5.9286	2.64471
Opposite-sex (*n* = 14)	5.3571	2.95107
**VALENCE**		
Same-sex (*n* = 14)	6.0000	2.03810
Opposite-sex (*n* = 14)	5.7143	1.77281

A one-way ANOVA was conducted comparing sex-typed participants' self-reported valence for high-salience same-sex avatars to their self-reported valence for high-salience opposite-sex avatars. Player-avatar sex match (same-sex × opposite-sex) was entered as a within-subject factor. Missing values were excluded listwise. There was no significant effect of player-avatar sex match on self-reported valence [*F*(_1, 13)_ = 0.331, *p* = 0.575, η^2^ = 0.025]. This suggests that SOTA had no significant impact on self-reported valence for sex-typed participants in the high-salience condition (see Table 2).

Together, the results of the two one-way ANOVAs suggest that for sex-typed participants in the high-salience condition the SOTA did not significantly affect their conscious (1) arousal or (2) valence. These findings are counter to the predictions made by Hypothesis 2.

#### H3

Hypothesis 3 stated that non-sex-typed participants' conscious (1) arousal and (2) valence would not differ significantly between the high-salience avatars of both sexes. To test both parts of this hypothesis, two separate one-way ANOVAs were calculated, one for arousal and one for valence.

A one-way ANOVA was calculated comparing non-sex-typed participants' self-reported arousal for high-salience male avatars to their self-reported arousal for high-salience female avatars. The SOTA (male × female) was entered as a within-subjects factor. Missing values were excluded listwise. There was no significant effect of SOTA on self-reported arousal [*F*_(1, 19)_ = 0.053, *p* = 0.821, η^2^ = 0.003]. This suggests that the SOTA had no impact on self-reported arousal for non-sex-typed participants in the high-salience condition (see Table 3).

**Table 3:** Means and standard deviations for self-reported arousal and valence for non-sex-typed participants in the high-salience condition.

**Condition**	***M***	***SD***
**AROUSAL**		
Same-sex (*n* = 20)	5.6500	2.41214
Opposite-sex (*n* = 20)	5.5500	2.41650
**VALENCE**		
Same-sex (*n* = 19)	5.7895	2.32329
Opposite-sex (*n* = 19)	5.6842	2.05623

A one-way ANOVA was calculated comparing non-sex-typed participants' self-reported valence for high-salience male avatars to their self-reported valence for high-salience female avatars. The SOTA (male × female) was entered as a within-subjects factor. Missing values were excluded listwise. There was no significant effect of SOTA on self-reported valence [*F*_(1, 18)_ = 0.100, *p* = 0.755, η^2^ = 0.006]. This suggests that the SOTA had no impact on self-reported valence for non-sex-typed participants in the high-salience condition (see Table 3).

The results of both one-way ANOVAs suggest that non-sex-typed participants' conscious (1) arousal and (2) valence did not differ significantly between the high-salience male avatar and high-salience female avatar. These findings provide some evidence for the predictions made by Hypothesis 3.

#### H4

Hypothesis 4 predicted that participants' subconscious (1) arousal and (2) valence would not differ significantly between low-salience male and low-salience female avatar conditions, regardless of sex-typing. To test both parts of this hypothesis, two 2 (avatar sex) × 2 (sex-type) × 90 (time) repeated measure ANOVAs were calculated, one for SC and one for corrugator activity.

**Figure 1:**
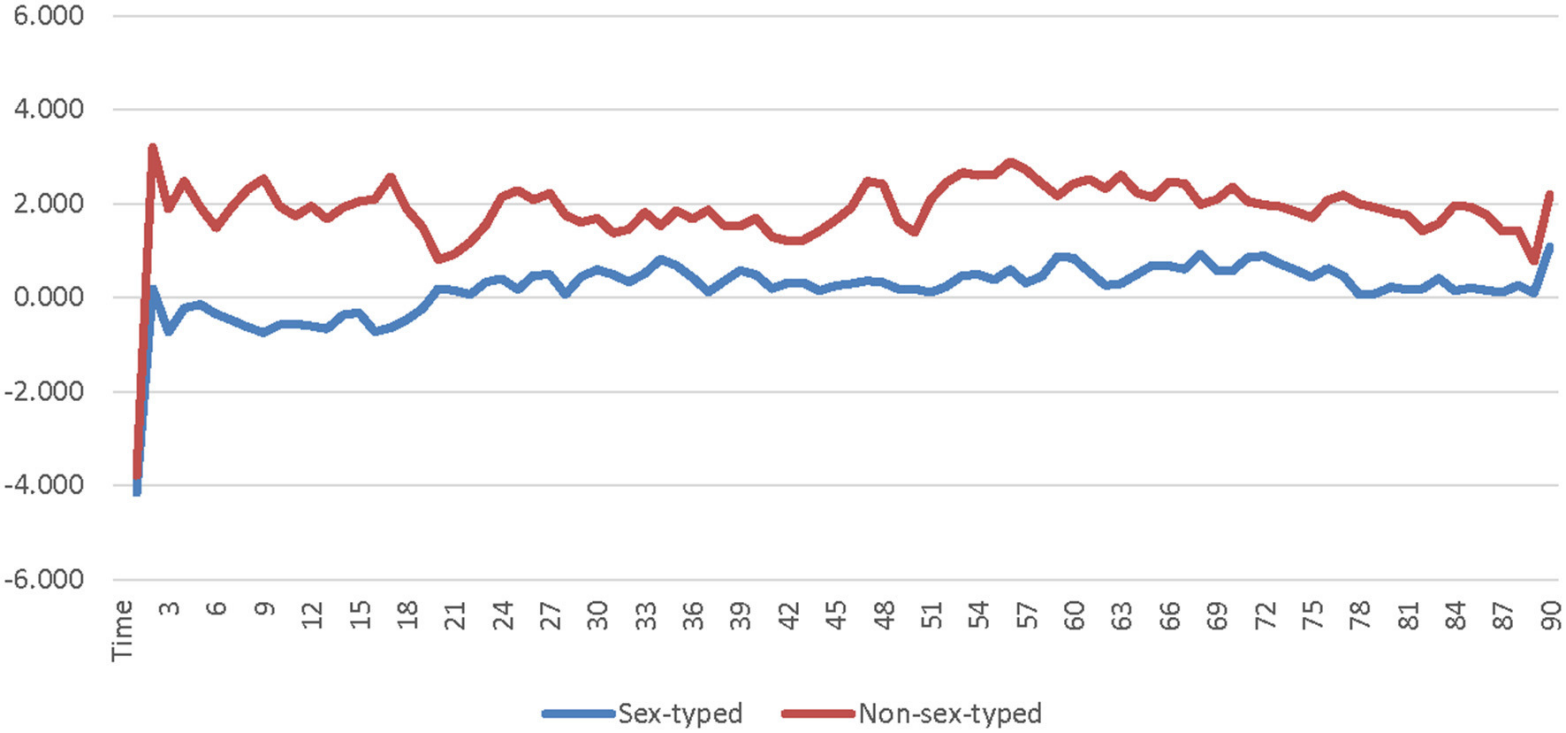
Sex-type by time interaction effect on skin conductance for the low-salience condition. This graph depicts the estimated marginal means for the interaction effect of sex-type by time on skin conductance in the low-salience condition.

**Figure 2:**
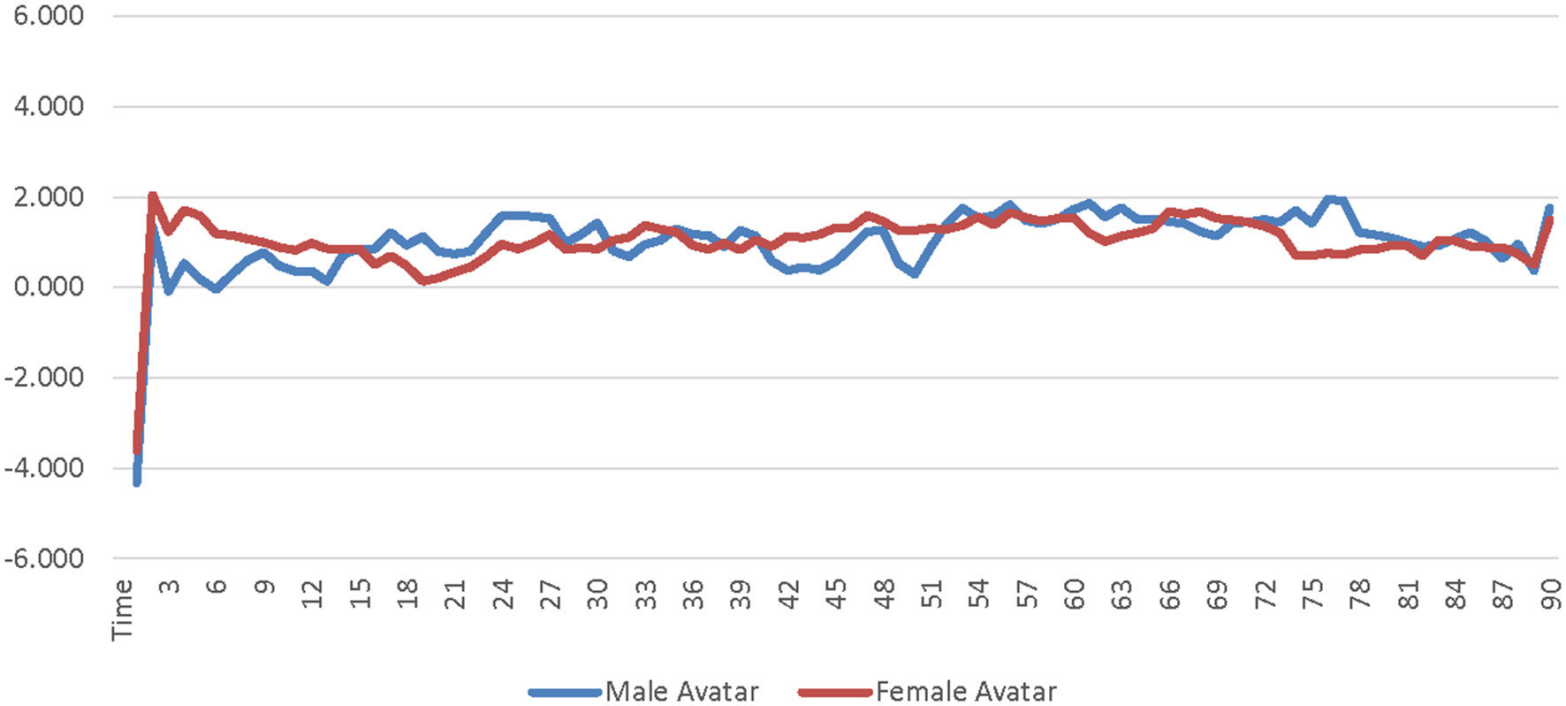
Avatar sex by time interaction effect on skin conductance for the low-salience condition. This graph depicts the estimated marginal means for the interaction effect of avatar sex by time on skin conductance in the low-salience condition.

**Figure 3:**
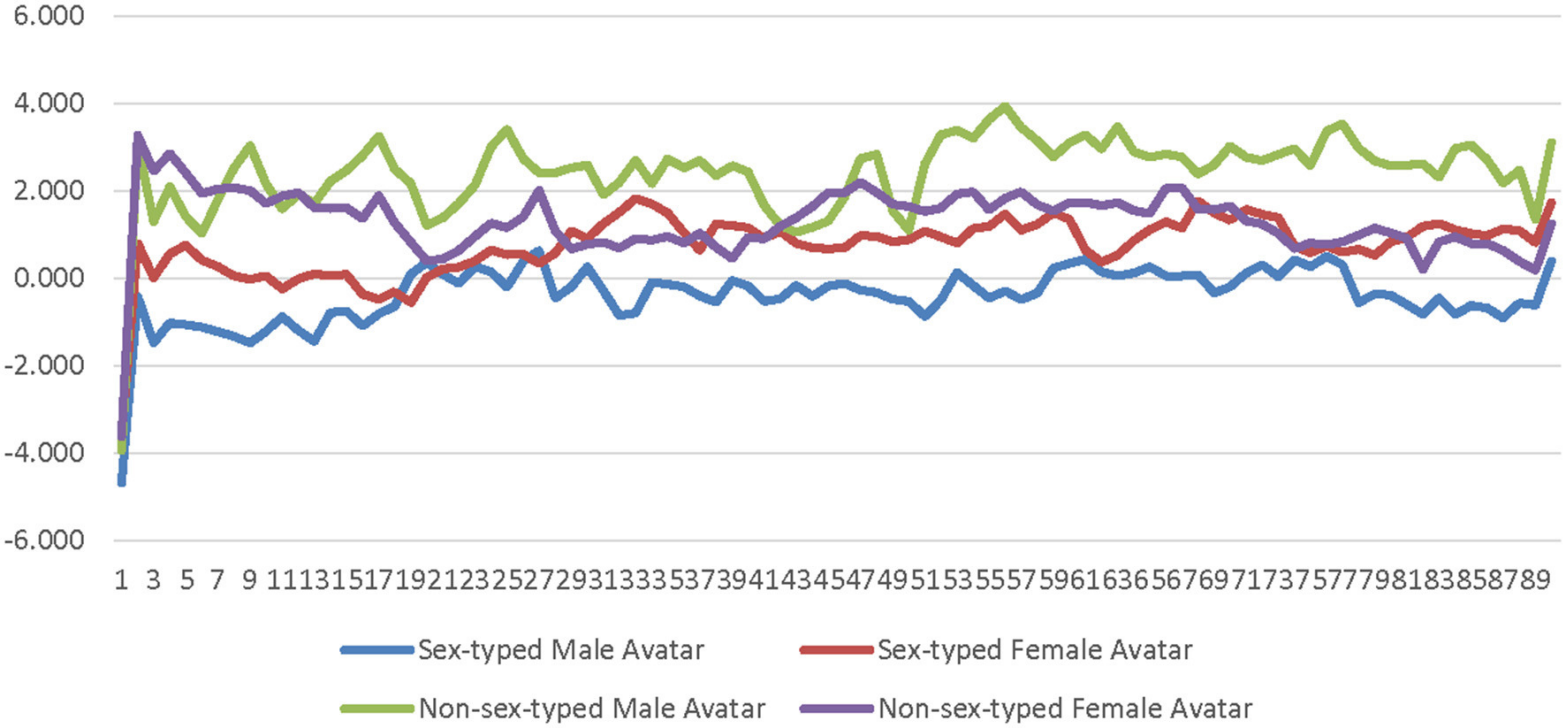
Sex-type by avatar sex by time interaction effect on skin conductance for the low-salience condition. This graph depicts the estimated marginal means for the interaction effect of sex-type by avatar sex by time on skin conductance in the low-salience condition.

A 2 (avatar sex) × 2 (sex-type) × 90 (time) repeated measures ANOVA was calculated comparing participants' SC for the low-salience male avatar to their SC for the low-salience female avatar. The SOTA (male × female) and time were both entered as within-subjects factors. Participant sex-type (sex-typed × non-sex-typed) was entered as a between-subjects factor. The main effect of SOTA was not significant [*F*_(1, 25)_ = 0.000309, *p* = 0.986, η_*p*_^2^ = 0.000012]; see Supplementary Table 1. The main effect of sex-type was significant [*F*_(1, 25)_ = 4.494, *p* = 0.044, η_*p*_^2^ = 0.152]. Non-sex-typed participants' SC (*M* = 1.869, *SE* = 0.631) was significantly higher than sex-typed participants' SC (*M* = 0.184, *SE* = 0.484) when using the low-salience male and female avatars (see Supplementary Table 2). The interaction of SOTA and sex-type was not significant [*F*_(1, 25)_ = 1.296, *p* = 0.266, η_*p*_^2^ = 0.049]. The interaction of time and sex-type was not significant [*F*_(89, 2, 225)_ = 0.922, *p* = 0.477, η_*p*_^2^ = 0.036] see Figure 1. The interaction of SOTA and time was not significant [*F*_(89, 2, 225)_ = 0.693, *p* = 0.631, η_*p*_^2^ = 0.027]; see Figure 2. The interaction of SOTA, sex-type, and time was not significant [*F*_(89, 2, 225)_ = 0.643, *p* = 0.669, η_*p*_^2^ = 0.025]; see Figure 3. These results suggest that SOTA and any of the interactions between the SOTA, participant sex-type, or time had no significant impact on participants' less conscious arousal. Additionally, these results suggest that participants' less conscious arousal for low-salience avatars differed depending on their sex-type such that non-sex-typed participants had significantly higher less conscious arousal while using the low-salience avatars than sex-typed participants.

**Figure 4:**
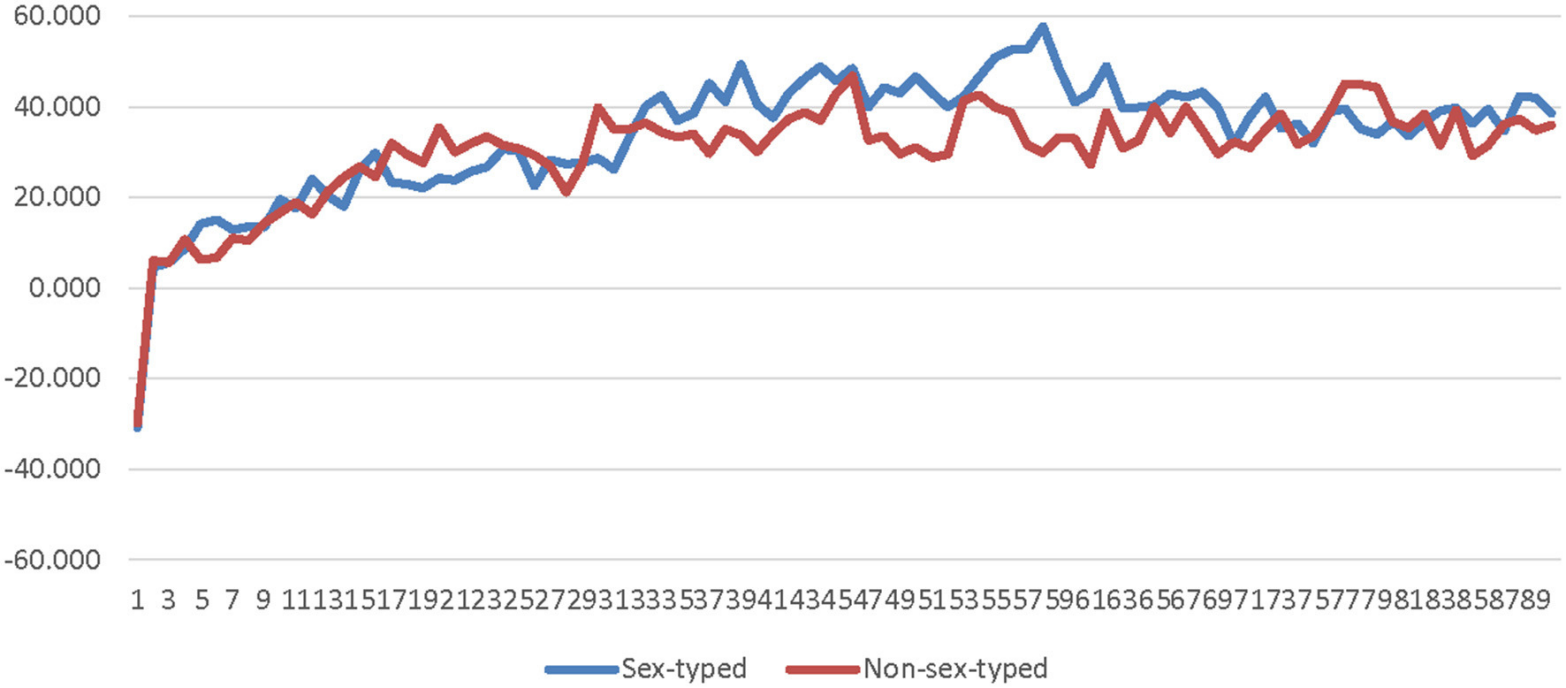
Sex-type by time interaction effect on corrugator activity for the low-salience condition. This graph depicts the estimated marginal means for the interaction effect of sex-type by time on corrugator activity in the low-salience condition.

**Figure 5:**
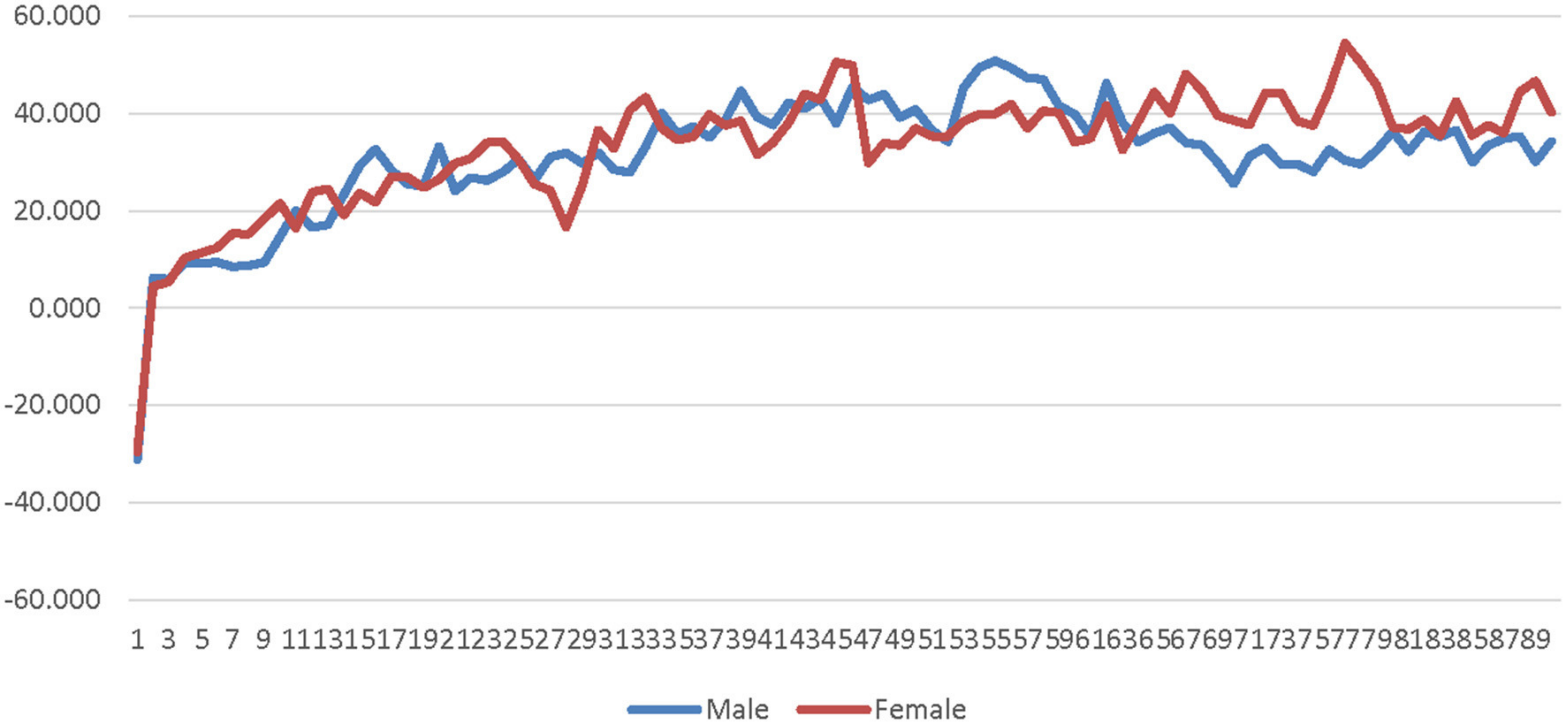
Avatar sex by time interaction effect on corrugator activity for the low-salience condition. This graph depicts the estimated marginal means for the interaction effect of avatar sex by time on corrugator activity in the low-salience condition.

**Figure 6:**
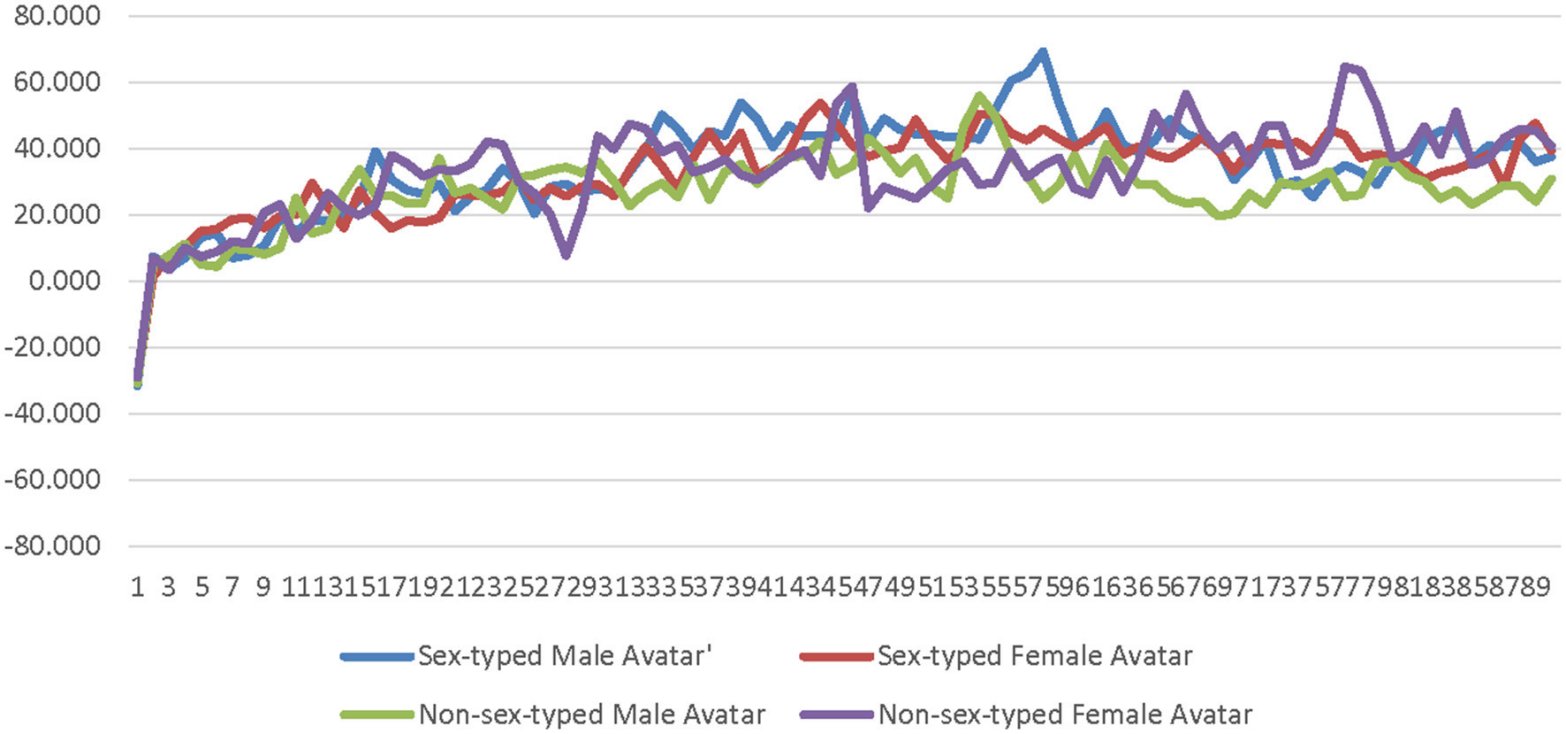
Sex-type by avatar sex by time interaction effect on corrugator activity for the low-salience condition. This graph depicts the estimated marginal means for the interaction effect of sex-type by avatar sex by time on corrugator activity in the low-salience condition.

A 2 (avatar sex) × 2 (sex-type) × 90 (time) repeated measures ANOVA was calculated comparing participants' corrugator activity for the low-salience male avatar to their corrugator activity for the low-salience female avatar. The SOTA (male × female) and time were both entered as within-subjects factors. Participant sex-type (sex-typed × non-sex-typed) was entered as a between-subjects factor. The main effect of SOTA was not significant [*F*_(1, 25)_ = 0.156, *p* = 0.696, η_*p*_^2^ = 0.006]; see Supplementary Table 1. The main effect of sex-type was not significant [*F*_(1, 25)_ = 0.086, *p* = 0.771, η_*p*_^2^ = 0.003]; see Supplementary Table 2. The interaction of SOTA and sex-type was not significant [*F*_(1, 25)_ = 0.552, *p* = 0.465, η_*p*_^2^ = 0.022]. The interaction of time and sex-type was not significant [*F*_(89, 2, 225)_ = 0.689, *p* = 0.692, η_*p*_^2^ = 0.027]; see Figure 4. The interaction of SOTA and time was not significant [*F*_(89, 2, 225)_ = 0.843, *p* = 0.589, η_*p*_^2^ = 0.033]; see Figure 5. The interaction of SOTA, sex-type, and time was not significant [*F*_(89, 2, 225)_ = 0.862, *p* = 0.571, η_*p*_^2^ = 0.033]; see Figure 6. These results suggest that the SOTA; participant sex-type; or any interactions of SOTA, participant sex-type, and time had no significant effect on participants' less conscious valence.

Taken together, the results of the two 2 (avatar sex) × 2 (sex-type) × 90 (time) repeated measures ANOVAs suggest that participants' less conscious (1) arousal and (2) valence did not significantly differ between the low-salience male and low-salience female avatar conditions, regardless of sex-type. Additionally, these analyses revealed that non-sex-typed participants' and sex-typed participants' less conscious arousal did significantly differ while using a low-salience avatar (of either sex), such that non-sex-typed participants experienced greater arousal than sex-typed participants while using low-salience avatars. These findings are partially in line with the predictions made by Hypothesis 4.

#### H5

Hypothesis 5 stated that sex-typed participants' subconscious (1) arousal and (2) valence would be higher for opposite-sex avatars than for same-sex avatars. To test both parts of this hypothesis, two 2 (player-avatar sex match) × 90 (time) repeated measure ANOVAs were calculated, one for SC and one for corrugator activity.

**Figure 7:**
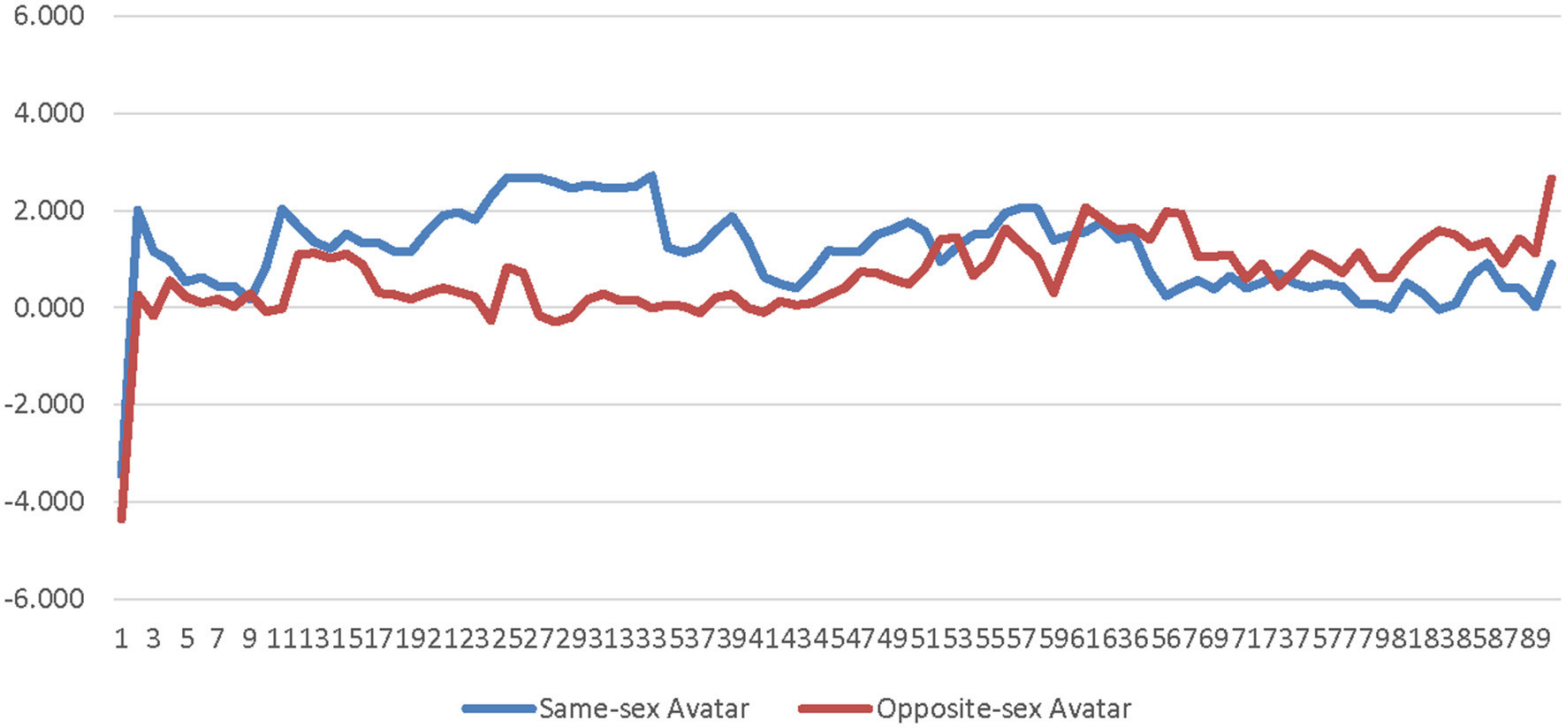
Avatar sex by time interaction effect on skin conductance for sex-typed participants in the high-salience condition. This graph depicts the estimated marginal means for the interaction effect of avatar sex by time on skin conductance for sex-typed participants in the high-salience condition.

A 2 (player-avatar sex match) × 90 (time) repeated measure ANOVA was conducted comparing sex-typed participants' SC for high-salience same-sex avatars to their SC for high-salience opposite-sex avatars. Player-avatar sex match (same-sex × opposite-sex) and time were entered as a within-subject factors. The main effect of player-avatar sex match was not significant [*F*_(1, 13)_ = 0.162, *p* = 0.694, η_*p*_^2^ = 0.012]. Additionally, the interaction of player-avatar sex match and time was not significant [*F*_(89, 1, 157)_ = 1.182, *p* = 0.325, η_*p*_^2^ = 0.083]. These results suggest that the SOTA had no impact on less conscious arousal for sex-typed participants in the high-salience condition (see Supplementary Table 3 and Figure 7).

**Figure 8:**
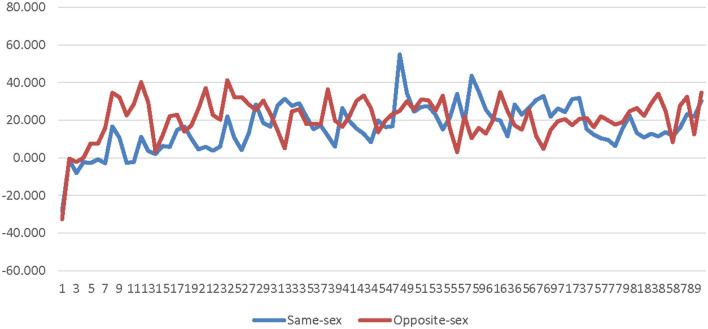
Avatar sex by time interaction effect on corrugator activity for sex-typed participants in the high-salience condition. This graph depicts the estimated marginal means for the interaction effect of avatar sex by time on corrugator for sex-typed participants in the high-salience condition.

A 2 (player-avatar sex match) × 90 (time) repeated measure ANOVA was conducted comparing sex-typed participants' corrugator activity for high-salience same-sex avatars to their corrugator activity for high-salience opposite-sex avatars. Player-avatar sex match (same-sex × opposite-sex) and time were entered as within-subject factors. The main effect of player-avatar sex match was not significant [*F*_(1, 13)_ = 1.126, *p* = 0.308, η_*p*_^2^ = 0.080]. Additionally, the interaction of player-avatar sex match and time was not significant [*F*_(89, 1, 157)_ = 0.999, *p* = 0.413, η_*p*_^2^ = 0.071]. These results suggest that the SOTA had no impact on less conscious valence for sex-typed participants in the high-salience condition (see Supplementary Table 3 and Figure 8).

Together, the results of the two 2 (player-avatar sex match) × 90 (time) repeated measure ANOVAs indicate that the SOTA did not significantly impact less conscious (1) arousal and (2) valence for sex-typed participants in the high-salience condition. These findings run counter to the predictions made by Hypothesis 5.

#### RQ1

Research Question 1 asked how non-sex-typed participants' embodied motivational processes of (1) emotional arousal and (2) emotional valence would be affected by an avatar with a highly salient sex. In order to test both parts of this research question, two 2 (avatar sex) × 90 (time) repeated measure ANOVAs were calculated, one for SC and one for corrugator activity.

**Figure 9:**
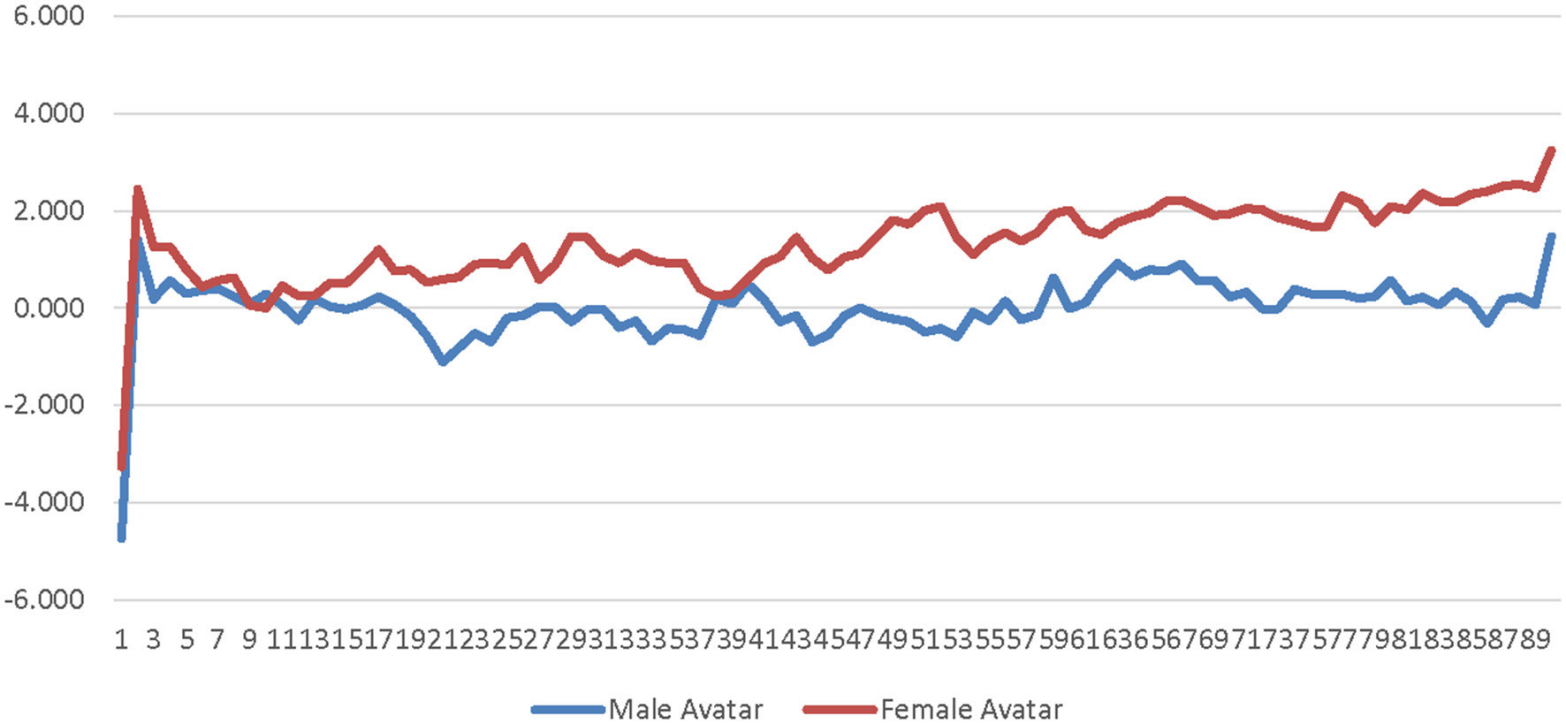
Avatar sex by time interaction effect on skin conductance for non-sex-typed participants in the high-salience condition. This graph depicts the estimated marginal means for the interaction effect of avatar sex by time on skin conductance for non-sex-typed participants in the high-salience condition.

A 2 (avatar sex) × 90 (time) repeated measure ANOVA was calculated comparing non-sex-typed participants' SC for the high-salience male avatar to their SC for the high-salience female avatar. The SOTA and time were both entered as within-subjects factors. The main effect of SOTA was not significant [*F*_(1, 19)_ = 1.969, *p* = 0.177, η_*p*_^2^ = 0.094]. The interaction effect of SOTA and time was also not significant [*F*_(89, 1, 691)_ = 0.834, *p* = 0.520, η_*p*_^2^ = 0.042]. Therefore, the SOTA does not appear to have an impact on less conscious arousal of non-sex-typed participants in the high-salience condition (see Supplementary Table 4 and Figure 9).

**Figure 10:**
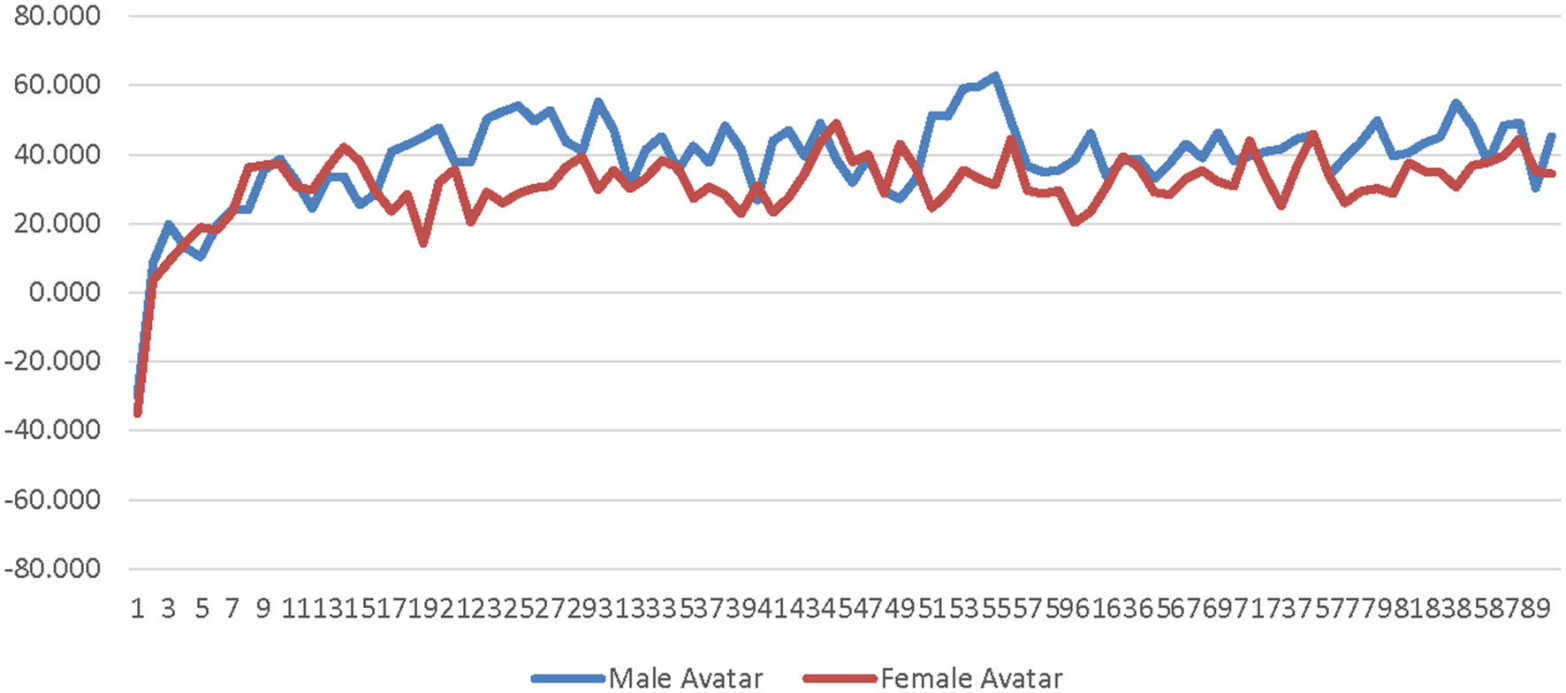
Avatar sex by time interaction effect on corrugator activity for non-sex-typed participants in the high-salience condition. This graph depicts the estimated marginal means for the interaction effect of avatar sex by time on corrugator for non-sex-typed participants in the high-salience condition.

A 2 (avatar sex) × 90 (time) repeated measure ANOVA was calculated comparing non-sex-typed participants' corrugator activity for the high-salience male avatar to their corrugator activity for the high-salience female avatar. The SOTA and time were both entered as within-subjects factors. The main effect of SOTA was not significant [*F*_(1, 19)_ = 1.155, *p* = 0.296, η_*p*_^2^ = 0.057]. The interaction effect of SOTA and time was also not significant [*F*_(89, 1, 691)_ = 0.927, *p* = 0.473, η_*p*_^2^ = 0.047]. Therefore, the SOTA did not appear to have an impact on the less conscious valence of non-sex-typed participants' in the high-salience condition (see Supplementary Table 4 and Figure 10).

Together, the results of the two 2 (avatar sex) × 90 (time) repeated measure ANOVAs suggest that for non-sex-typed participants in the high-salience condition, neither their subconscious arousal nor their subconscious valence were influenced by the SOTA.

## Discussion

The objective of this study was to investigate the effects of the SOTA, salience of the SOTA, and participant sex-type on less conscious embodied emotional arousal and valence and consciously perceived emotional arousal and valence. It was predicted that the SOTA would have a threshold effect on conscious and less conscious arousal and valence such that when the salience of the SOTA was low the SOTA would have no significant effect on individuals' conscious and less conscious emotional arousal and valence, regardless of their sex-type (H1, H4); but, when the salience of the SOTA was high the SOTA would have significant effects on individuals' conscious and less conscious emotional arousal and valence based on their sex-type (H2, H3, H5, RQ1). Specifically, it was predicted that when the salience of the SOTA was high, sex-typed individuals' conscious and less conscious emotional arousal and valence would differ depending on the SOTA (H2, H5), and that non-sex-typed individuals' conscious emotional arousal and valence would not differ, regardless of the SOTA (H3). No specific predictions were made regarding the effects of the SOTA when the salience of the SOTA was high on non-sex-typed individuals' less conscious emotional arousal and valence (RQ1).

The results of the analyses conducted to test these predictions were almost entirely non-significant. Further interpretation of the non-significant findings is difficult because the analyses were underpowered. Despite having a sample that met the recommendations of the power analysis (i.e., the power analysis indicated the minimum necessary sample size was 78 and our final sample was 81) the number of participants in the individual cells was relatively small (e.g., there were only 14 sex-typed participants in the high-salience condition), and thus it is unclear whether the non-significant results are due to the small cell sizes or because no effect actually exists. Despite this there are still useful insights to be learned from the results of this study.

Contrary to the prediction that there would be no differences between sex-typed and non-sex-typed participants' less conscious emotional arousal when the salience of the SOTA was low (H4), it was found that non-sex-typed participants' SC was significantly higher than sex-typed participants' when using low-salience avatars. The effect size associated with this finding is large, which is surprising because based on previous research on the salience of the SOTA, it was expected that sex-typed and non-sex-typed participants' responses to low sex-salience avatars would not differ (Coyle and Liben, 2016). However, in contrast to this previous research the findings of this study suggest individuals' sex-type may influence their responses to low sex-salience avatars in certain ways. Additionally, SC is an indicator of the strength of the activation of the motivational systems (i.e., arousal; Potter and Bolls, 2012). Sex-typed participants' SC was lower when using low-salience avatars compared to non-sex-typed participants', suggesting that low-salience avatars may be more motivationally relevant to non-sex-typed individuals than sex-typed individuals. According to GST, sex-typed individuals are much more likely to process information in terms of their gender schemas (Bem, 1981b). Because the low-salience avatars lacked explicit gender-cues, they may have been a-schematic to sex-typed participants, and thus less motivationally relevant, which in turn resulted in sex-typed participants' SC for low-salience avatars being lower than non-sex-typed participants'. In other words, it might not be that non-sex-typed individuals find low-salience avatars more motivationally relevant than sex-typed individuals, but that low-salience avatars have such little motivational relevance to sex-typed individuals that their physiological reactions to low-salience avatars are lower than those of non-sex-typed individuals.

### Limitations

The present study has several limitations that should be kept in mind when interpreting its results.

First, as previously mentioned, the analyses of the study were underpowered. There are a number of reasons that may have caused the analyses to be underpowered despite having a sample size that met the requirements of the previously conducted power analysis.

One possibility is that the effect size of the SOTA on conscious and less conscious emotional arousal and valence is much smaller than initially predicted. The original power analysis was conducted for a small effect size (Cohen's *f* of 0.18; Cohen, 1988); however, it is possible that the effect of the SOTA on conscious and less conscious emotional arousal and valence is much smaller. If this was the case, then it is possible that an effect might be found with a larger sample size. However, the majority of the effect sizes for the non-significant findings in the study were either small or below small, which suggests that the effect of the SOTA on conscious and less conscious emotional arousal and valence may be so small as to simply be inconsequential regardless of the sample size. In other words, while increasing the sample size may increase the study's statistical power, it would not likely change the results in a meaningful way.

Another factor that potentially led to the analyses being underpowered was the use of the short form of the BSRI. According to the *Bem Sex Role Inventory Professional Manual* the short form of the BSRI tends to classify more people as androgynous and feminine than the long form due to the social desirability of the feminine items retained on the short form (Bem, 1981a). Because sex-type is determined based on individuals' biological sex and gender identity (Bem, 1981b), this can lead to more individuals being categorized as non-sex-typed and cross-typed when using the short form of the BSRI than when using the long form (Bem, 1981a). The short form of the BSRI used in this study may have led to an increased number of participants being categorized as non-sex-typed and cross-sex-typed, which would directly impact the size of the cells used for the analyses because they were based on participants' sex-type and salience condition (i.e., it may have led to non-sex-typed cells being larger and sex-typed cells being smaller because participants who would be categorized as sex-typed by the long form were categorized as non-sex-typed or cross-typed by the short form). Thus, the use of the short form of the BSRI may have potentially contributed to the small cell sizes and caused the analyses to be underpowered. The short form of the BSRI was chosen over the long form as a matter of practicality. It consists of only 30 items compared to the long form, which consists of 60 items; thus, the short form was chosen in the interest of keeping experimental sessions under an hour to avoid participant fatigue (Bem, 1981a).

Another issue that may have contributed to the analyses being underpowered was the unanticipated number of participants classified as cross-sex-typed (e.g., biological female with a masculine gender identity). According to the *Bem Sex Role Professional Manual*, cross-sex-typed individuals normally only make up a small percent of individuals in a given sample (12% of males and 12% of females; Bem, 1981a). Based on those percentages and the number of male and female participants in the final sample for this study, it was expected that approximately 8–10 participants would be classified as cross-sex-typed. However, while the percent of female participants classified as cross-sex-typed was as expected (11%, *n* = 5), the number of cross-sex-typed males in the present sample was well above 12%. Of the 36 male participants, 41% (*n* = 15) were classified as cross-sex-typed. Even accounting for how the short form of the BSRI increases the number of cross-sex-typed classifications, this is still an unexpectedly high number of cross-sex-typed individuals. Thus, of the 81 participants in the final sample, 20 participants were classified as cross-sex-typed. Because cross-sex-typed participants were excluded from analyses, the high number of cross-sex-typed individuals in the sample may have negatively impacted the cell sizes. Future studies may want to consider the use of a screener questionnaire in order to avoid having too many participants of one sex-type.

A second limitation of this study is that no manipulation check was carried out for the salience of the SOTA. The purpose of this experiment was to investigate potential effects of a common intrinsic feature of avatars, their sex. This feature can objectively vary for avatars based on avatar design. This variance in avatar design makes features that define the SOTA more or less visible or explicit as an intrinsic feature of the avatar used to play a game. This variance, defined by intrinsic features of an avatar, was manipulated in this experiment. There has been some past discussion of the necessity of manipulation checks in experiments on media processes and effects (O'Keefe, 2003; Tao and Bucy, 2007). These authors advance the argument that manipulation checks on independent variables in experiments are not appropriate when the independent variable is an intrinsic feature of the media stimulus that is manipulated based on production/design of the stimulus. Based on these arguments and because we manipulated concrete intrinsic features of our stimuli (e.g., the avatars' attires) we did not conduct a manipulation check. However, although the armor used in the low-salience condition was very similar for both male and female avatars it did not fully eliminate secondary sex traits, and thus could have influenced the results of our study. Future research should consider conducting a manipulation check to confirm salience of the SOTA was successfully manipulated.

A third limitation of this study is that we did not control for any non-salience-based effects of the outfits the avatars wore (i.e., the armor and the rags). Previous research into the Proteus Effect has found that avatar characteristics, like their attire, can influence how their users feel and behave (Yee and Bailenson, 2007; Ratan et al., 2020). These studies would suggest that the armor worn by the low-salience avatars could cause participants in the low-salience condition to feel more powerful/strong and that the rags used in the high-salience condition may have caused participants to less powerful/weak. Feeling more or less powerful could potentially have affected participants conscious and less conscious feelings of arousal, and thus influenced the results. Future research should conduct pre-tests of the avatars used as stimuli in order to control for any non-salience-based effects on the dependent variables.

A fourth limitation of the study is that the sample used for the study was a volunteer sample drawn from a student participant pool. Generalizing the findings of this study to other populations should be done cautiously as previous research has found that student samples can differ from non-student samples in important ways (Peterson, 2001).

A fifth and final limitation is that participants encountered significant lag during certain sections of game while playing it, even though the game's graphics settings had been properly set for the hardware capabilities of the computer it was played on. The lag participants experienced may have caused frustration, which may have influenced conscious and less conscious emotional arousal and valence. Future studies should use more powerful computers or consider using a dedicated gaming console for the gameplay portion of the study in order to ensure a smooth experience for participants.

### Implications and Future Research

The results of this study found that participants' sex-type had a large effect on their less conscious arousal in response to low-salience avatars, such that non-sex-typed participants' less conscious arousal was significantly higher when using these avatars than sex-typed participants. This suggests that game developers may want to consider the salience of the SOTA when creating their games. Because individuals' less conscious arousal is indicative of how strongly their motivational systems (i.e., the appetitive and the aversive systems) are active, variation in less conscious arousal caused by the salience of the SOTA could potentially impact the way they experience a game. Thus, developers that are seeking to ensure that their game is experienced similarly by all players may want to avoid using low-salience avatars. Instead, developers may want to design avatars that are androgynous (i.e., have both explicit masculine *and* feminine cues) or abstract. Future research should explore more deeply the way that sex-type impacts individuals' conscious and less conscious responses to avatars with low sex-salience in order to determine the exact nature of this relationship.

Additionally, the present project had a number of limitations that constrained the insights that could be drawn from its results. However, a brief glance at the directionality of the means associated with the analyses conducted suggests that with adequate statistical power the results may have differed in many ways from our initial predictions. Therefore, conducting a replication of the study with a larger sample could provide important insights into the effects of the SOTA, the salience of the SOTA, and sex-type on players' embodied emotional processing of video games and their conscious emotional responses to the gaming experience.

As video games continue to grow in popularity and people spend increasingly more time with their avatars it, becomes important for us to understand how avatars can affect players' processing of and responses to video games. Studies such as the present one play an important role in providing a foundation upon which future studies of the effects of avatars on players' processing and responses to games can build and expand.

## Data Availability Statement

The raw data supporting the conclusions of this article will be made available by the authors, without undue reservation.

## Ethics Statement

The studies involving human participants were reviewed and approved by The Human Research Protection Program at Texas Tech University. The patients/participants provided their written informed consent to participate in this study.

## Author Contributions

DP wrote the manuscript, designed and ran the experiment, conducted the analysis, and interpreted the results. PB acted as academic advisor for the project, contributed to the abstract and introduction, and assisted with the data analysis and interpretation. All authors contributed to the article and approved the submitted version.

## Conflict of Interest

The authors declare that the research was conducted in the absence of any commercial or financial relationships that could be construed as a potential conflict of interest.

## Footnotes

^1^One of the core assumptions of the LC4MP is that the brain processes all stimuli as “real” regardless of whether they are mediated or not (Lang, 2009; Fisher et al., 2018). In other words, the brain reacts to an attacking tiger on TV the same way it reacts to a “real” attacking tiger. This means that “survival needs” in a video game will be prioritized and processed the same way they would in the “real world.” Thus, just like in the real world if you are in a dangerous situation (e.g., a gunfight) what your body looks like is likely not top of mind. You may be hyper aware of your body in terms of its positioning and/or whether it's been injured, but you're not thinking about whether or not your body is biologically male/female. You are simply focused on keeping that body from getting hurt.

^2^It should be noted that a number of the above predictions predict null effects; while it is not common practice to predict null effects in some academic disciplines, in the context of the present study, it was felt that the predictions of null effects were justified because of the study's focus on examining conscious and less conscious mental processes across multiple conditions and contexts. Specifically, that in order to most precisely and accurately describe the expected effects of the SOTA on participants' conscious and less conscious emotional arousal and valence across each of the different conditions, it was necessary for several of the hypotheses to predict null effects.

^3^Participants' gender was calculated using the “hybrid method” described in the *Bem Sex Role Inventory Professional Manual* (Bem, 1981a). Of the 81 participants the majority were categorized as feminine (50.6%, *n* = 41), followed by androgynous (23.5%, *n* = 19), undifferentiated (13.6%, *n* = 11), and masculine (12.3%, *n* = 10). The median masculinity score was 4.7 (*M* = 4.8, *SD* = 0.82) and the median femininity score was 5.3 (*M* = 5.3, *SD* = 0.88). Missing data were replaced using the series mean.

^4^Bem (1981b) argues that the BSRI possesses a “number of features” which make it ideal for identifying individuals' sex-types, and that the findings of previous research that has used the BSRI to determine participants' sex-types provide convergent validity for the use of the measure in this manner. Based on Bem's (1981b) arguments, as well as the fact that we are not aware of any other measure of sex-type we chose to use the BSRI following the procedures outlined by Bem (1981b).
