# Supplementary material for: The Effects of Sex-Type, the Sex of the Avatar, and Salience of the Sex of the Avatar on Emotional Valence and Arousal
Source: Front Psychol. 2021 May 10;12:659547. doi: 10.3389/fpsyg.2021.659547 (PMC8141742; doi:10.3389/fpsyg.2021.659547)
Supplement: Supplementary file 1 [file Data_Sheet_1.docx]

Supplementary Materials

**
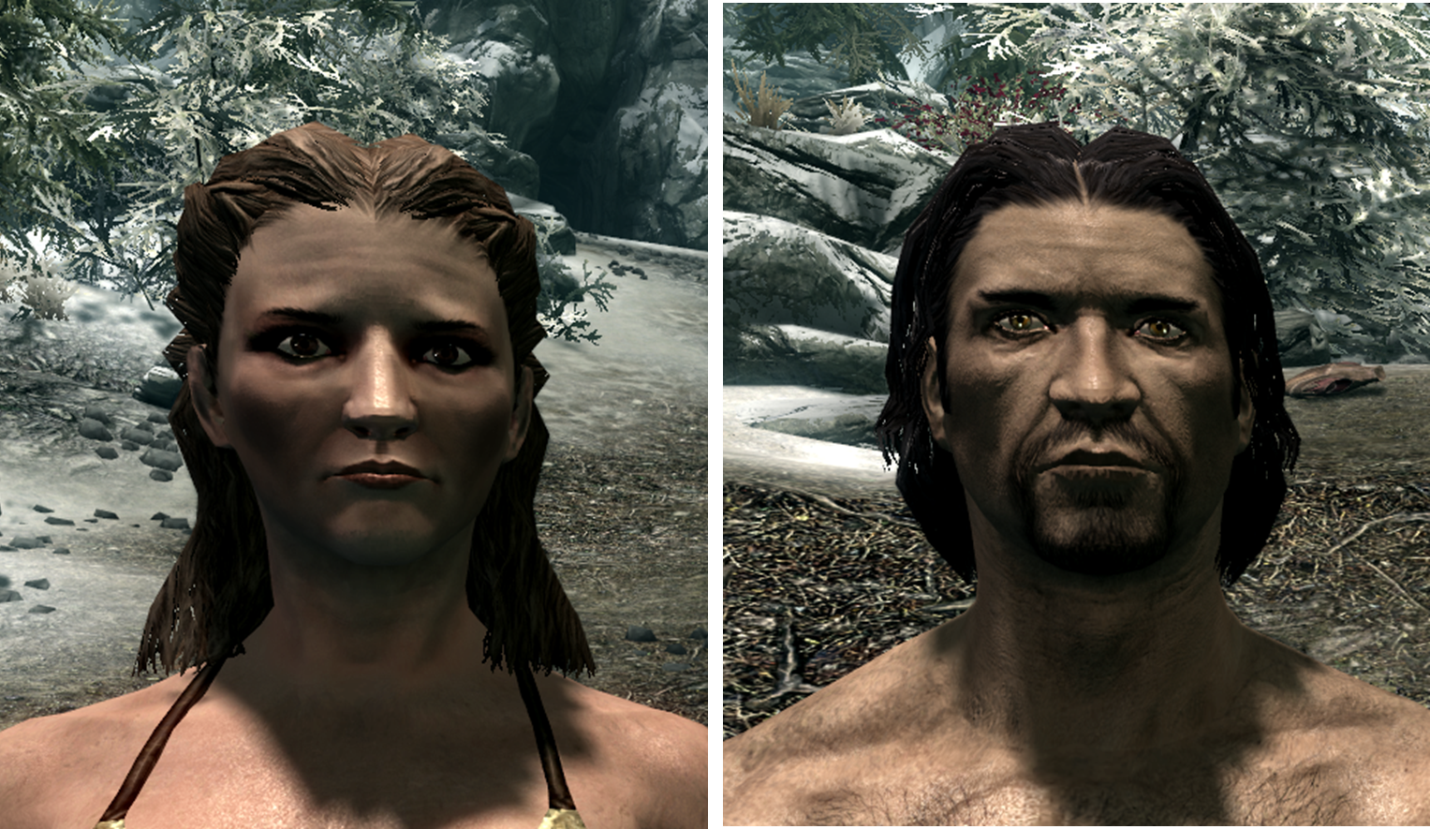
**

**Supplementary Figure 1.** Default avatars. This figure presents screenshots of the default female (left) and male (right) avatars for the Imperial race in *The Elder Scrolls V: Skyrim*.

**
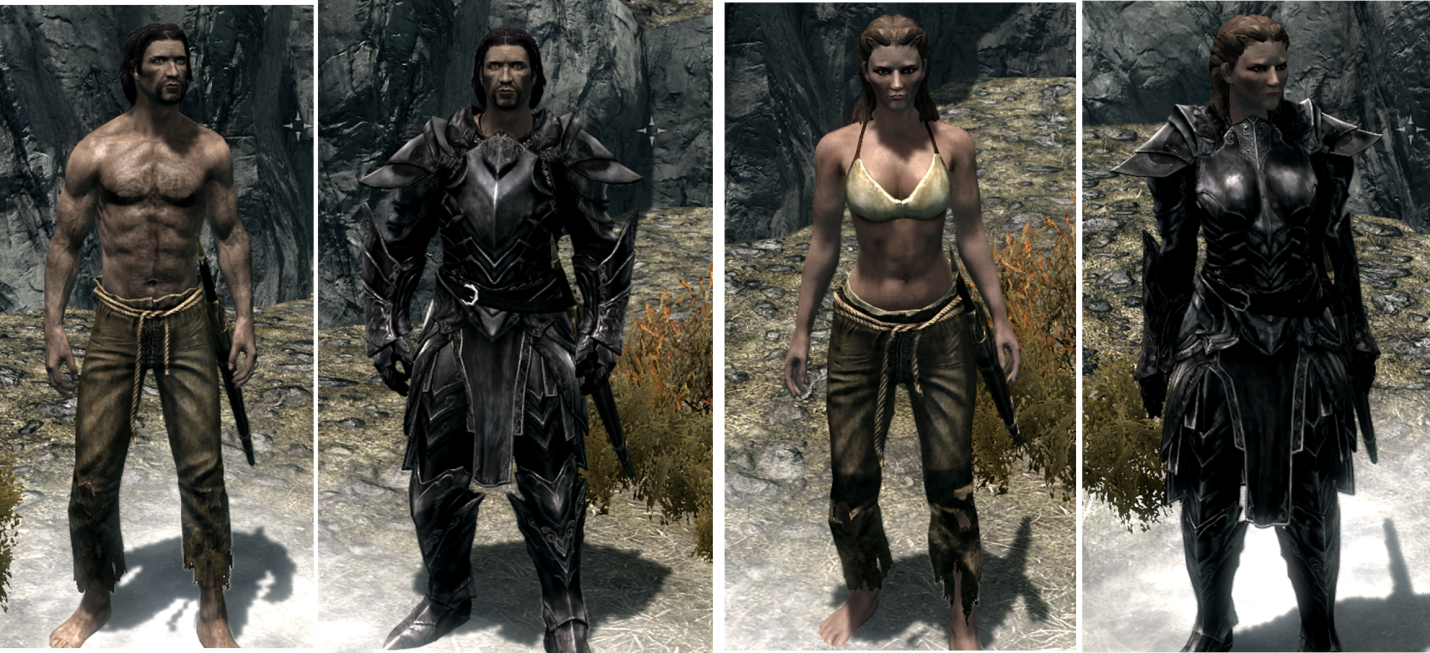
**

**Supplementary Figure 2.** Avatar attire. This figure presents screenshots of the ragged trousers and ebony armor items as they appear on the default male (left) and default female (right) avatars for the Imperial race in *The Elder Scrolls V: Skyrim*.

**Supplementary Table 1.** Estimated Marginal Means and Standard Error for the Main Effect of Avatar Sex on Skin Conductance and Corrugator Activity in the Low Salience Condition

|  | SC |  | Corrugator Activity |
| --- | --- | --- | --- |

| Condition | *EMM* | *SE* |  | *EMM* | *SE* |
| --- | --- | --- | --- | --- | --- |
| Male Avatar | 1.018 | 0.624 |  | 31.194 | 7.137 |
| Female Avatar | 1.035 | 0.642 |  | 33.212 | 5.817 |

**Supplementary Table 2.** Estimated Marginal Means and Standard Error for the Main Effect of Sex-type on Skin Conductance and Corrugator Activity in the Low Salience Condition

|  | SC | |  | Corrugator Activity | |
| --- | --- | --- | --- | --- | --- |
| Condition | *EMM* | *SE* |  | *EMM* | *SE* |
| Sex-typed | 0.184 | 0.484 |  | 33.963 | 7.290 |
| Non-sex-typed | 1.869 | 0.631 |  | 30.442 | 9.505 |

**Supplementary Table 3.** Estimated Marginal Means and Standard Error for the Main Effect of Avatar Sex on Skin Conductance and Corrugator Activity for Sex-typed Participants in the High Salience Condition

|  | SC | |  | Corrugator Activity | |
| --- | --- | --- | --- | --- | --- |
| Condition | *EMM* | *SE* |  | *EMM* | *SE* |
| Same-sex Avatar | 1.151 | 0.778 |  | 16.405 | 10.069 |
| Opposite-sex Avatar | 0.643 | 0.756 |  | 20.933 | 11.117 |

**Supplementary Table 4.** Estimated Marginal Means and Standard Error for the Main Effect of Avatar Sex on Skin Conductance for Non-sex-typed Participants in the High Salience Condition

|  | SC | |  | Corrugator Activity | |
| --- | --- | --- | --- | --- | --- |
| Condition | *EMM* | *SE* |  | *EMM* | *SE* |
| Male Avatar | 0.004 | 0.697 |  | 38.922 | 12.118 |
| Female Avatar | 1.324 | 0.605 |  | 30.844 | 9.533 |
